# Changes in Patient Experiences and Assessment of Gaming Among Large Clinician Practices in Precursors of the Merit-Based Incentive Payment System

**DOI:** 10.1001/jamahealthforum.2021.3105

**Published:** 2021-10-08

**Authors:** Eric T. Roberts, Zirui Song, Lin Ding, J. Michael McWilliams

**Affiliations:** 1 Department of Health Policy and Management, University of Pittsburgh Graduate School of Public Health, Pittsburgh, Pennsylvania; 2 Department of Health Care Policy, Harvard Medical School, Boston, Massachusetts; 3 Department of Medicine, Massachusetts General Hospital, Boston, Massachusetts; 4 Division of General Internal Medicine and Primary Care, Brigham and Women’s Hospital, Boston, Massachusetts

## Abstract

**Importance:**

Medicare’s Merit-Based Incentive Payment System (MIPS), a public reporting and pay-for-performance program, adjusts clinician payments based on publicly reported measures that are chosen primarily by clinicians or their practices. However, measure selection raises concerns that practices could earn bonuses or avoid penalties by selecting measures on which they already perform well, rather than by improving care—a form of gaming. This has prompted calls for mandatory reporting on a smaller set of measures including patient experiences.

**Objective:**

To examine (1) practices’ selection of Consumer Assessment of Healthcare Providers and Systems (CAHPS) patient experience measures for quality scoring under the pay-for-performance program and (2) the association between mandated public reporting on CAHPS measures and performance on those measures within precursor programs of the MIPS.

**Design, Setting, and Participants:**

This cross-sectional study included 2 analyses. The first analysis examined the association between the baseline CAHPS scores of large practices (≥100 clinicians) and practices’ selection of these measures for quality scoring under a pay-for-performance program up to 2 years later. The second analysis examined changes in patient experiences associated with a requirement that large practices publicly report CAHPS measures starting in 2014. A difference-in-differences analysis of 2012 to 2017 fee-for-service Medicare CAHPS data was conducted to compare changes in patient experiences between large practices (111-150 clinicians) that became subject to this reporting mandate and smaller unaffected practices (50-89 clinicians). Analyses were conducted between October 1, 2020, and July 30, 2021.

**Main Outcomes and Measures:**

The primary outcomes of the 2 analyses were (1) the association of baseline CAHPS scores of large practices with those practices’ selection of those measures for quality scoring under a pay-for-performance program; and (2) changes in patient experiences associated with a requirement that large practices publicly report CAHPS measures starting in 2014.

**Results:**

Among 301 large practices that publicly reported patient experience measures, the mean (IQR) age of patients at baseline was 71.6 (70.4-73.2 ) years, and 55.8% of patients were women (IQR, 54.3%-57.7%). Large practices in the top vs bottom quintile of patient experience scores at baseline were more likely to voluntarily include these scores in the pay-for-performance program 2 years later (96.3% vs 67.9%), a difference of 28.4 percentage points (95% CI, 9.4-47.5 percentage points; *P* = .004). After 2 to 3 years of the reporting mandate, patient experiences did not differentially improve in affected vs unaffected practices (difference-in-differences estimate: −0.03 practice-level standard deviations of the composite score; 95% CI, −0.64 to 0.58; *P* = .92).

**Conclusions and Relevance:**

In this cross-sectional study of US physician practices that participated in precursors of the MIPS, large practices were found to select measures on which they were already performing well for a pay-for-performance program, consistent with gaming. However, mandating public reporting was not associated with improved patient experiences. These findings support recommendations to end optional measures in the MIPS but also suggest that public reporting on mandated measures may not improve care.

## Key Points

**Question:**

Do clinician practices game pay-for-performance programs by selectively reporting measures on which they already perform well, and does mandating public reporting on patient experience measures improve care?

**Findings:**

In this cross-sectional analysis of patient experience data from Consumer Assessment of Healthcare Providers and Systems (CAHPS) surveys, practices were more likely to voluntarily include CAHPS measures in a Medicare pay-for-performance program when they previously scored higher on these measures. However, mandatory public reporting of CAHPS measures was not associated with improved patient experiences with care.

**Meaning:**

These findings support calls to end voluntary measure selection in public reporting and pay-for-performance programs, including Medicare’s Merit-Based Incentive Payment System, but also suggest that requiring practices to report on patient experiences may not produce gains.

## Teaser

This cross-sectional study examines health care practices’ selection of patient experience measures for quality scoring under the pay-for-performance program and the association between mandated public reporting and performance on those measures within precursor programs of the Medicare Merit-Based Incentive Payment System.

## Introduction

The Medicare Merit-Based Incentive Payment System (MIPS) is the largest public reporting and pay-for-performance program for clinicians.^1,2,3^ The MIPS was introduced in 2017 and scores approximately 880 000 clinicians on 4 domains of care (quality, clinical practice improvement, use of interoperable health information technology, and cost), which are publicly reported on Medicare’s Physician Compare website.^2,4^ Clinicians receive an overall performance score, calculated as a weighted average of performance across these domains, which determines whether they receive positive, negative, or neutral payment adjustments 2 years later.^5,6^

Performance in the MIPS is assessed primarily from measures selected by clinicians or their practices. For example, practices can report any 6 of nearly 300 measures in the quality domain, which accounts for nearly half of the overall performance score.^5,7^ This flexibility was intended to promote broad participation in the MIPS and stimulate quality improvement in diverse clinical settings. However, allowing practices to select the measures they report raises concerns. First, measure selection undermines a central goal of public reporting: to compare clinicians and practices on a consistent set of measures.^8,9^ Second, because scoring in the MIPS is based on relative performance (ie, how a practice compares with others on a given measure), practices have a strong incentive to select measures on which they expect to perform well relative to other practices.^9,10,11,12^ Thus, measure selection raises concerns about gaming because practices may be able to earn bonuses or avoid penalties by choosing measures on which they already score well, rather than by investing time and resources to provide better care.^11,13^

Citing these concerns, the Medicare Payment Advisory Commission recommended replacing the MIPS with a program focused on a smaller set of mandatory performance measures, including patient experiences with care.^8^ Such a change would effectively make public reporting mandatory for a core set of measures and eliminate practices’ ability to select which measures affect payment adjustments. However, it remains unclear whether practices select measures on which they already score well, and if mandating public reporting on patient experience measures may be beneficial.

To address these questions, we studied precursors of the MIPS—the Value-Based Payment Modifier (VM) and Physician Quality Reporting System (PQRS)—the configurations of which enabled us to examine practice measure selection and mandatory public reporting.^14,15^ We conducted 2 analyses focused on patient experience measures from the Consumer Assessment of Healthcare Providers and Systems (CAHPS) survey. First, we studied measure selection among large practices (≥100 clinicians), which were required to publicly report CAHPS measures starting in 2014 but could voluntarily include these measures in a separate pay-for-performance program.^16,17,18,19^ We examined the association between practices’ baseline CAHPS scores and subsequent selection of these measures for quality scoring in the pay-for-performance program. Second, we used the introduction of the CAHPS reporting mandate for large practices and a difference-in-differences design to examine the relationship between mandatory public reporting and changes in patient experiences with care.^18,19,20^

## Methods

This cross-sectional study was approved by the Harvard Medical School Committee on Human Studies, which granted a waiver of informed consent because we analyzed deidentified secondary data. We followed the Strengthening the Reporting of Observational Studies in Epidemiology (STROBE) reporting guidelines for cohort studies.^21^ Analyses were conducted between October 1, 2020, and July 30, 2021.

### Policy Context

We studied 2 programs that were direct predecessors of the MIPS: the Value-Based Payment Modifier (VM), a pay-for-performance program, and the Physician Quality Reporting System (PQRS), a public reporting program.

The VM was introduced as a voluntary program in 2013 and was fully phased in as a mandatory program for practices with 10 or more clinicians by 2015.^19,20^ Under the VM, practices received upward, downward, or neutral payment adjustments based on their performance in 2 areas: quality of care and per-patient spending. Practices were evaluated on a mandatory set of quality and spending measures but could select additional measures, including CAHPS measures, that contributed to an overall quality score.^16^ A prior study examined the association between pay-for-performance incentives in the VM and performance on the program’s mandatory measures.^14^ Here, we examined whether practices included CAHPS measures as an optional component of their overall VM quality score.

The PQRS was introduced in 2007 and initially paid bonuses to practices that voluntarily reported performance measures, a subset of which were displayed online on Physician Compare.^15^ Over time, components of the PQRS became mandatory.^20^ Beginning in 2014, large practices with 100 or more clinicians were required to publicly report CAHPS measures to avoid penalties.^18,19^ Because compliance with this reporting mandate grew rapidly after the first year, we omitted 2014 as a transition year and examined changes in patient experiences from 2011 to 2013 to 2015 to 2016. Additional details about the VM and PQRS are in eTable 1 of the Supplement.

Practices that participated as ACOs in the Medicare Shared Savings Program (MSSP) were not initially included in the VM and reported patient experiences through a separate CAHPS for ACOs survey.^16^ Therefore, we excluded practices that participated in the MSSP in any year from 2012 (the program’s first year) through 2016. The MIPS replaced the VM and PQRS in 2017 and made public reporting of patient experiences optional for all practices.^22^

### Data Sources and Study Population

#### Measure Selection and Gaming

We analyzed practice-level data using 2014 to 2016 VM Practice Files, which included the CAHPS scores of large practices that reported these measures for the PQRS. These scores were calculated by CMS based on responses to the CAHPS for PQRS survey, which was administered by a third party to individuals with traditional (ie, fee-for-service) Medicare. Medicare beneficiaries were sampled from practices where they received most primary care.^17^ We observed annual practice-level scores for 11 patient experience domains and whether practices elected to include these scores in their overall VM quality score (practices could include all or none of the domain scores^23^).

Practices were informed of their CAHPS scores after these measures could have contributed to the practice’s annual VM quality score.^24^ To assess how practice decisions to include CAHPS scores in the VM changed over time as practices learned about their prior performance, we studied a sample of practices that reported CAHPS measures in 2016 (the last year of the VM and PQRS) and started reporting them in 2014 or 2015.

#### Changes in Patient Experiences Associated With Mandatory Public Reporting

We analyzed patient-level data from the fee-for-service Medicare CAHPS survey, which is separate from but closely related to the CAHPS for PQRS survey (eTable 2 in the Supplement).^16^ The fee-for-service Medicare CAHPS survey is administered annually to representative samples of community-dwelling Medicare beneficiaries.^25,26^ Because the survey is conducted early each year and asks respondents to rate their care over the prior 6 months, we analyzed surveys administered from 2012 to 2014 and 2016 to 2017 to assess patient experiences in 2011 to 2013 and 2015 to 2016, respectively, omitting the 2015 survey pertaining to the 2014 transition year.

We used linked Medicare claims from the year before the survey to attribute each respondent to the practice (identified by its taxpayer identification number) that accounted for the plurality of the respondent’s office visits with primary care clinicians in that year.^27,28^ We excluded 25.7% of respondents without primary care claims and 8.9% of respondents for whom practice size could not be determined. Of remaining respondents, we excluded 36.0% whose practices participated in the MSSP in any year from 2012 to 2016. We limited each annual sample to patients attributed to practices with 50 to 89 or 111 to 150 clinicians, excluding practices with 90 to 110 clinicians to mitigate attenuation bias from small year-to-year fluctuations in practice size that could have changed exposure to the reporting mandate.

### Outcome Variables

In analyses of measure selection, our primary outcome was an indicator that a practice elected to include CAHPS scores in its overall VM quality score. In analyses of mandatory public reporting, we examined patient experience measures from 5 domains of the fee-for-service Medicare CAHPS survey that corresponded to domains from the CAHPS for PQRS survey: rating of primary physician, physician communication, timely access to care, access to specialists, and care coordination (eTable 2 in the Supplement). We also analyzed a composite patient experience score, which we calculated as an equally weighted average of scores for the 5 domains.

### Practice Size

We measured practice size as the number of clinicians billing under a TIN, consistent with how CMS defined practice size for the VM and PQRS. In analyses of measure selection by large practices, we identified practices with 100 or more clinicians from practice sizes reported in the VM Practice File. In analyses of public reporting, we measured annual practice size using Medicare Provider Practice and Specialty (MD-PPAS) files.^29^ We measured practice size in the year prior to the fee-for-service Medicare CAHPS survey to align with the period for which we attributed patients to practices.

### Respondent Variables

Analyses of the fee-for-service Medicare CAHPS survey included respondents’ demographic characteristics (eg, age, sex, and race and ethnicity), health status, and proxies for socioeconomic status (enrollment in Medicaid and the Medicare Savings Programs^30^). Race and ethnicity were assessed from the RTI race variable, which classifies Medicare beneficiaries’ race and ethnicity based on Social Security Administration data and an imputation algorithm that identifies additional Hispanic and Asian beneficiaries.^31^ Variable descriptions are in section 5 in the Supplement.

### Statistical Analyses

#### Measure Selection and Gaming

We categorized large practices that reported CAHPS measures for the PQRS into quintiles of their baseline performance, defined as an equally weighted average of scores on 11 patient experience domains in the first year that practices reported CAHPS measures for the PQRS (2014 or 2015 in our sample). We compared the proportion of practices that included CAHPS scores, measured in the baseline year and up to 2 years later, in their annual VM quality scores, across quintiles of baseline scores. To examine the relationship between baseline scores and future performance, we calculated correlations of practice-level scores across years. Analyses were conducted using SAS statistical software (version 9.4; SAS Institute, Inc).

#### Changes in Patient Experiences Associated With Mandatory Public Reporting

We conducted a difference-in-differences analysis to assess changes in patient experiences associated with mandatory public reporting. Specifically, we compared changes in patient experiences from a preintervention period (2011-2013) to a postintervention period (2015-2016) between large practices (111-150 clinicians) affected by the reporting mandate and smaller unaffected practices (50-89 clinicians). For each patient experience score, we estimated a linear difference-in-differences model:

*E*(*Score_i,t,k,c,h_*) = β_0_ + β_1_*LargePractice_k_* + β_2_(2015 or 2016)*_t_* + β_3_*LargePractice_k_* × (2015 or 2016)*_t_* + β_4_*X_i,t_* + β_5_*MA_c,t_* + *year_t_* + *HRR_h_*

where *Score_i,t,k,c,h_* is a score for respondent *i* in year *t* who was attributed to practice *k* and lived in county *c* and Hospital Referral Region *h; LargePractice_k_* indicates that practice *k* had 111 to 150 clinicians; and (2015 or 2016)*_t_* denotes the postintervention period. We adjusted for respondents’ health, demographic, and socioeconomic characteristics (*X_it_*), to account for any compositional changes among patients of large vs smaller practices over time. We adjusted for the annual Medicare Advantage rate by county (*MA_c,t_*) to control for potential spillovers of the Medicare Advantage program onto fee-for-service Medicare beneficiaries^32,33^; year fixed effects (*year_t_*) to control for time trends; and hospital referral region fixed effects (*HRR_h_*) to control for time-invariant market factors affecting care for patients across large and smaller practices.

Thus, β_3_ represents the adjusted within-HRR differential change in patient experiences associated with mandatory public reporting for large practices (pooled across HRRs), through 2 to 3 years after the mandate’s introduction. To facilitate interpretation, we reported estimates of β_3_ scaled by the practice-level standard deviations (SDs) of preintervention period scores (termed effect sizes). We adjusted for survey weights and used robust variance estimation to account for clustering within practices. Section 5 in the Supplement provides additional information about these analyses and interpretation of estimates. The threshold for statistical significance was *P* < .05 using 2-sided tests.

To examine practice compliance with the reporting mandate, we assessed rates of public reporting of CAHPS measures among large vs smaller practices during 2015 to 2016, which we compared with rates in 2014. We also conducted 2 tests of assumptions underlying the difference-in-differences design.^34^ First, we estimated differential changes in patient- and practice-level characteristics between large and smaller practices before and after 2014.^35^ The absence of differential changes supports the assumption that the difference-in-differences model isolates changes in patient experiences associated with public reporting from compositional changes among practices or their patients. Second, we compared preintervention trends in patient experience scores between large and smaller practices. Similar preintervention trends support the assumption that differences in scores between large and smaller practices would have remained constant had the reporting mandate for large practices not been introduced.^34^

#### Sensitivity Analyses

We conducted 2 sensitivity analyses. First, we examined changes in patient experiences from concurrent CAHPS surveys. Second, we reestimated our difference-in-differences models on a broader sample of survey respondents, whom we attributed to practices based on office visits with primary care clinicians or specialists.

## Results

### Measure Selection and Gaming

Among practices with 100 or more clinicians, 301 publicly reported CAHPS measures in 2016 and started reporting them in 2014 or 2015 (742 practice-years). At baseline (2014 or 2015), these practices had a mean of 431 clinicians and 10 229 patients enrolled in fee-for-service Medicare, among whom the mean age was 71.6 years, 55.8% were women, and 17.8% also received Medicaid (eTable 6 in the Supplement). In 492 (66.3%) of these practice-years, practices voluntarily included CAHPS scores in their overall VM quality score.

**Figure 1.**
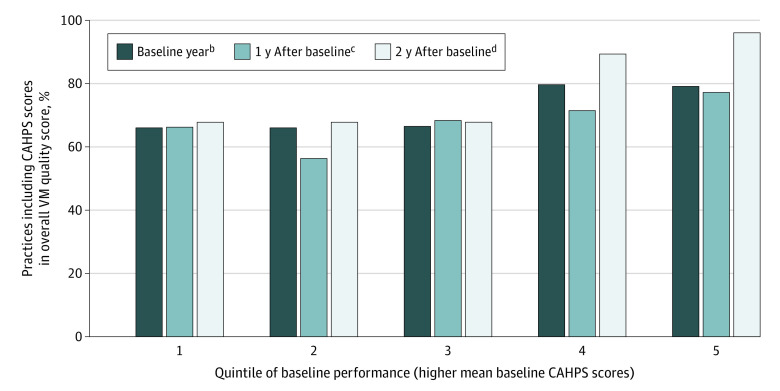
Proportions of Practices Including CAHPS Patient Experience Scores in the VM, by Quintile of Mean Baseline Scores^a^ CAHPS Indicates Healthcare Providers and Systems; VM, Value-Based Payment Modifier. ^a^Among 301 large physician practices (≥100 clinicians) that publicly reported CAHPS patient experience measures in 2016 (the last year of the Physician Quality Reporting System [PQRS] and VM) and started reporting them in either 2014 (140 practices) or 2015 (161 practices). Practices were categorized into quintiles of their baseline performance, which we calculated as an equally weighted average of scores on 11 patient experience domains in the first year a practice reported CAHPS measures for the PQRS (2014 or 2015). Practice-level scores in these 11 domains were reported by CMS in the VM Practice File. ^b^Percentage of practices voluntarily including CAHPS patient experience measures, assessed in the baseline year, in their overall VM quality score in the baseline year. ^c^Percentage of practices voluntarily including CAHPS measures, assessed 1 year after baseline, in their overall VM quality score 1 year after baseline. ^d^Percentage of practices voluntarily including CAHPS measures, assessed 2 years after baseline, in their overall VM quality score two years after baseline.

Of the 60 practices in the highest quintile of baseline CAHPS scores, 78.9% elected to include these scores in their overall VM quality score for the baseline year, vs 65.9% in the lowest baseline quintile, a difference of 13.0 percentage points (95% CI, 3.9-29.8 percentage points; *P* = .13; Figure 1). Two years later, 96.3% of practices in the highest baseline quintile included these CAHPS measures in the VM, vs 67.9% of practices in the lowest baseline quintile, a difference of 28.4 percentage points (95% CI, 9.4-47.5 percentage points; *P* = .004). In the subset of practices that first reported CAHPS measures in 2014, the 60 practices in the lowest baseline quintile became less likely to include CAHPS measures by 2016, whereas practices in the highest baseline quintile became more likely to include CAHPS measures by 2016 (eFigure 1 in the Supplement). Overall, CAHPS scores were positively correlated within practices across years (eTable 7 in the Supplement).

### Changes in Patient Experiences Associated With Mandatory Public Reporting

We analyzed a sample of 21 738 respondents to fee-for-service Medicare CAHPS surveys administered from 2012 to 2014 and 2016 to 2017 (eFigure 2 in the Supplement). In the preintervention period, the mean age of respondents in large practices was 74.1 years, 56.8% of respondents were female, and 6.3% were enrolled in Medicaid (Table 1), closely resembling the community-dwelling Medicare population in the CAHPS sampling frame.^26,36^ We found few meaningful differential changes in respondent characteristics between large vs smaller practices from the preintervention to postintervention periods (Table 1; eFigure 3 in the Supplement), and no statistically significant differential changes in practice characteristics (eTable 8 in the Supplement).

**Table 1.** Before Intervention Characteristics and Changes Among Survey Respondents in Large and Smaller Physician Practices

Respondent characteristics^a^	Before intervention (2011-2013), %		Change from before to after intervention (2011-2013 to 2015-2016)			
	Large practices (111-150 clinicians) [n = 4351 respondents in 344 practices]^b^^,^^c^	Smaller practices (50-89 clinicians) [n = 9399 respondents in 945 practices]^b^^,^^c^	%		Differential change^d^	
			Large practices (111-150 clinicians)	Smaller practices (50-89 clinicians)	Estimate (95% CI)	*P* value
Age, y	74.1	74.0	−0.3	−0.3	0.0 (−0.7 to 0.7)	.99
Sex						
Female	56.8	59.1	1.1	−2.4	3.5 (0.6 to 6.5)	.02
Male	43.2	40.9	−1.1	2.4	−3.5 (−6.5 to −0.6)	.02
Race and ethnicity						
Asian	1.1	0.9	0.1	0.3	−0.2 (−1.0 to 0.6)	.62
Black	4.6	5.5	2.2	0.1	2.1 (−2.8 to 7.0)	.39
Hispanic	2.7	2.6	−0.3	0.4	−0.7 (−2.1 to 0.6)	.28
White	89.3	89.4	−3.2	−2.2	−1.0 (−6.4 to 4.4)	.72
Other^e^	2.4	1.6	1.2	1.4	−0.2 (−2.0 to 1.6)	.81
Disabled^f^	16.1	14.7	−1.7	1.8	−3.5 (−6.2 to −0.8)	.01
End-stage bladder disease	0.6	0.6	0.2	−0.1	0.3 (−0.2 to 0.8)	.18
HCC score^g^	1.3	1.3	0.0	0.1	−0.1 (−0.1 to 0.0)	.08
CCW chronic conditions, number^h^	6.6	6.6	0.1	0.1	0.0 (−0.3 to 0.2)	.71
Enrolled in Medicaid^i^	6.3	6.2	1.1	−0.1	1.2 (−1.4 to 3.9)	.36
Enrolled in a Medicare Savings Program^j^	3.5	3.7	−0.2	0.2	−0.4 (−1.7 to 0.9)	.56
Education^k^						
Less than high school	11.5	11.8	−2.7	−2.7	0.0 (−2.3 to 2.4)	.98
High school graduate	31.1	31.7	−4.1	−3.6	−0.5 (−4.0 to 2.9)	.77
Some college	27.2	26.5	0.0	1.0	−1.0 (−4.1 to 2.0)	.51
College graduate	11.3	10.7	2.1	1.7	0.4 (−2.1 to 2.8)	.78
Graduate education	15.6	15.3	3.5	3.5	0.0 (−2.9 to 3.0)	.98
Current smoker	9.0	9.1	−1.5	−0.5	−1.0 (−2.8 to 0.8)	.28
Use of helper to complete survey	8.0	8.5	0.1	0.8	−0.7 (−2.5 to 1.0)	.42
Any functional limitations^l^	5.2	5.6	0.6	−0.7	1.3 (0.0 to 2.6)	.04
Self-reported general health score^m^	3.1	3.1	0.1	0.1	0.0 (−0.1 to 0.1)	.60
Self-reported mental health score^m^	3.7	3.7	0.0	0.0	0.0 (−0.1 to 0.1)	.97

Abbreviation: CCW, Chronic Condition Data Warehouse; HCC, Hierarchical Condition Category.

^a^ Characteristics of respondents to the 2012 to 2014 and 2016 to 2017 fee-for-service Medicare Healthcare Providers and Systems (CAHPS) surveys, as assessed from the survey and Medicare enrollment and claims data from the year prior to the survey (unless otherwise noted). Respondents attributed to practices where they received the majority of primary care visits in the year prior to the survey. All estimates adjusted for CAHPS survey weights. Respondent characteristics for categorical variables are reported as percentages, which allows us to appropriately display survey-weighted distributions of patient characteristics in the population, rather than numerical frequencies which would not incorporate survey weighting.

^b^ Practice size calculated as the number of unique clinicians that billed under a practice’s taxpayer identification number in the year prior to the survey.

^c^ Number of respondents and practices in the preintervention period (2011-2013). In the postintervention period (2015-2016), the sample included 2989 respondents in 262 large practices (111-150 clinicians) and 4999 respondents in 635 smaller practices (50-89 clinicians). Across the preintervention and postintervention periods, the sample included 21 738 respondents in 2186 practices.

^d^ That is, changes in the mean or proportion of patients with the characteristic shown in the table row between large and smaller practices from the preintervention to the postintervention periods as defined in the table columns. We estimated these differential changes by fitting a respondent-level linear difference-in-differences model for each characteristic as a function of a postintervention period indicator, an indicator that a patient’s practice had 111 to 150 clinicians, and an interaction between these indicators. Differential changes are given by the regression coefficient on the interaction term. The 95% CIs and *P* values were calculated using robust standard errors clustered by practice (taxpayer identification number).

^e^ Category consists of beneficiaries whose race and ethnicity were classified as American Indian, Alaska Native, or Other in the Medicare beneficiary summary file.

^f^ Disability was original reason for Medicare entitlement.

^g^ Hierarchical Condition Category (HCC) scores constructed from Medicare beneficiaries’ demographic characteristics in the year prior to the survey and diagnoses claims from 2 years prior to the survey. Higher HCC scores indicate higher predicted spending in the following year.

^h^ Count of chronic conditions from the Medicare Chronic Condition Data Warehouse (CCW), which draws from claims since 1999 to measure the presence of 27 chronic diseases among Medicare beneficiaries. We assessed the presence of chronic conditions reported on claims prior to the survey year.

^i^ That is, enrollment in full Medicaid.

^j^ Enrollment in 1 of the Medicare Savings Programs, which are partial Medicaid benefits that pay for Medicare Part B premiums, and in some cases Parts A and B cost sharing, for Medicare beneficiaries with low incomes and assets (Section 5 in the Supplement).

^k^ A small proportion (approximately 4%) of respondents did not report their education. We retained these observations in regression analyses by including an indicator variable for missing education status.

^l^ Proportion reporting difficulty with 1 or more activities of daily living (bathing, dressing, eating, using chairs, walking, and using the toilet).

^m^ Assessed on a scale of 1 to 5, where 1 indicates poor self-rated health or mental health and 5 indicates excellent self-rated general or mental health.

From 2014 to 2015 to 2016, the proportions of large and smaller practices that reported patient experience measures increased by 36.7 and 4.4 percentage points, respectively, constituting a differential increase among large practices of 32.3 percentage points (95% CI, 23.6-41.0 percentage points; *P* < .001; eFigure 4 in the Supplement).

**Figure 2.**
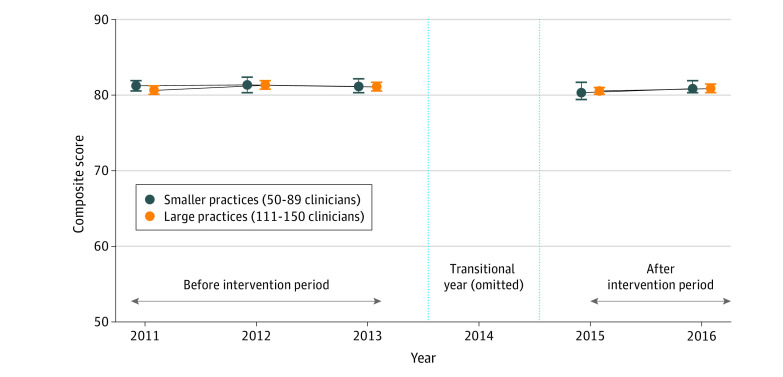
Mean Annual Composite Patient Experience Scores in Large vs Smaller Practices Plotted are unadjusted mean composite scores reflecting patient experiences with care from 2011 to 2013 and 2015 to 2016 in large practices (111-150 clinicians) and smaller practices (50-89 clinicians). Patient experiences with care in 2011 to 2013 and 2015 to 2016 assessed from the 2012 to 2014 and 2016 to 2017 fee-for-service Medicare Healthcare Providers and Systems surveys, respectively. We omitted the 2015 survey, pertaining to patient experiences in 2014, as a transitional year. Practice size was calculated as the number of unique clinicians that billed under a practice’s taxpayer identification number in the year prior to the survey. Scores are standardized to a 0 to 100 scale, with higher scores representing better patient experiences with care (Section 3 in the Supplement). Error bars represent 95% CIs for annual mean scores and were calculated using robust standard errors clustered by practice (taxpayer identification number). The unadjusted difference-in-differences estimate was −0.19 points of the composite score, equivalent to −0.12 practice-level standard deviations (SDs) of the composite score (95% CI, −0.73 to 0.50 SDs; *P* = .72).

Patient experiences did not differentially improve in large vs smaller practices from the preintervention period to 2 to 3 years after the public reporting mandate (Figure 2). The adjusted estimate for the differential change in the composite patient experience score was −0.03 practice-level SDs of the score (95% CI, −0.64 to 0.58; *P* = .92; Table 2). Results were similar in analyses of individual domain scores (Table 2) and in sensitivity analyses (eTables 9 and 10 in the Supplement). Preintervention trends in scores were comparable between large and smaller practices (eFigure 5 in the Supplement).

**Table 2.** Difference-in-Differences Estimates for Association Between Mandatory Public Reporting and Patient Experiences With Care

Patient experience scores^a^	Before intervention (2011-2013)		Difference-in-differences estimates^d^		
	Mean scores among large practices (111-150 clinicians)^b^	SDs of practice scores^c^	Regression estimate	Effect size, SDs (95% CI)^e^^,^^f^	*P* value^f^
Composite score^g^	80.6	1.6	−0.05	−0.03 (−0.64 to 0.58)	.92
Domain-specific scores^h^					
Rating of primary physician	90.1	1.0	−0.17	−0.16 (−1.12 to 0.79)	.74
Physician communication	88.2	1.3	0.19	0.15 (−0.68 to 0.97)	.73
Timely access to care	66.2	2.3	−0.24	−0.10 (−0.55 to 0.34)	.65
Access to specialists	84.8	2.5	0.95	0.38 (−0.49 to 1.25)	.39
Care coordination	80.4	1.5	0.48	0.32 (−0.38 to 1.01)	.37

Abbreviations: CAHPS, Consumer Assessment of Healthcare Providers and Systems; HRR, Hospital Referral Region; MA, Medicare Advantage.

^a^ Patient experiences with care assessed from the fee-for-service Medicare CAHPS survey. We used responses to surveys administered from 2012 to 2014 and 2016 to 2017 to assess patient experiences with care from 2011 to 2013 and 2015 to 2016, respectively. We omitted the 2015 survey, pertaining to patient experiences in 2014, as a transitional year.

^b^ Mean scores among practices with 111 to 150 clinicians in the preintervention period, adjusted for respondent characteristics in Table 1, annual county-level MA penetration rates, HRR fixed effects, year fixed effects, and survey weights. Scores are standardized to a 0 to 100 scale, with higher scores representing better patient experiences with care (Section 3 in the Supplement).

^c^ Standard deviation (SD) of the practice-level distribution of patient experience scores, estimated among all practices in the preintervention period (Section 5 in the Supplement for details of this calculation).

^d^ Difference-in-differences estimates represent the differential change in composite or domain-specific patient experience scores between large practices (111-150 clinicians) and smaller practices (50-89 clinicians) from the preintervention period (2011-2013) to the postintervention period (2015-2016), adjusted for respondent characteristics in Table 1 and survey weights.

^e^ Effect sizes are difference-in-differences estimates scaled by the practice-level SD of each score. An effect size of −0.16 SDs is equivalent to the difference between the median practice (50th percentile) and a practice at the 44th percentile of performance. The corresponding 95% CIs are also scaled by the practice-level SD in each score.

^f^ 95% CIs and *P* values were calculated using robust standard errors clustered by practice (taxpayer identification number).

^g^ Calculated at the patient level as an equally weighted average of items comprising all domain-specific scores (Section 3 in the Supplement).

^h^ Calculated at the patient level as an equally weighted average (at the patient level) of items within each domain-specific score (Section 3 in the Supplement).

## Discussion

In this study of US clinician practices that participated in precursors of the MIPS, we found that large practices were more likely to select CAHPS measures for quality scoring under pay-for-performance when the practices had previously scored well on these measures. However, mandatory public reporting on CAHPS measures was not associated with improved performance on these measures after 2 to 3 years.

The patterns of measure selection we detected were consistent with gaming because a practice would have increased its chances of receiving a bonus or avoiding a penalty by selecting measures on which it expected to perform well, independent of actual quality improvement efforts. For practices, prior CAHPS scores constituted a reliable signal of their future performance, given the high correlation of their scores across years. Accordingly, we found that practices with higher initial CAHPS scores became more likely to include these measures in the pay-for-performance program over time, consistent with strategic measure selection informed by knowledge of prior performance. This evidence underscores concerns about gaming in the MIPS, where practices have even greater latitude to select performance measures that affect payment adjustments.

Therefore, the findings of this study support recommendations to end measure selection in the MIPS.^8^ Measure selection undermines a central goal of public reporting, which is to facilitate comparisons across practices on the same measures, and is wasteful to the extent that it enables practices to earn bonuses or avoid penalties without improving care.^9,11,37^ Our findings add to evidence about how practices strategically respond to voluntary components of pay-for-performance programs. For example, in an analysis of the first year of the VM, when practices could voluntarily receive payment adjustments tied to performance, Joynt and colleagues^38^ found that practices with better performance scores were more likely to accept performance-based payment incentives.

However, our results also suggest that mandating public reporting of patient experience measures, as recommended in some MIPS reform proposals,^8^ may not improve care. Our finding that patient experiences did not improve under mandatory public reporting is consistent with other studies of public reporting programs for clinicians and hospitals, which found little change in quality of care, reflected in process and health outcome measures, under these programs.^39,40,41,42,43^ To our knowledge, no prior studies of public reporting programs examined performance on patient experiences measures. These measures capture aspects of care that are reported directly by patients and may be more comprehensible to patients than technical aspects of care (eg, process measures), underscoring their importance as quality indicators.^44^

These findings remain salient amid forthcoming changes to the MIPS. In 2022, CMS will launch the MIPS Value Pathways framework. This framework is intended to reduce reporting burdens by defining core sets of measures, pertaining to specific conditions or specialties, that practices can report. However, MIPS Value Pathways framework may not eliminate gaming, since CMS has indicated that practices will be able to choose which measures (from a given measure set) they report.^45^

### Limitations

Our study had some limitations. First, the extent to which practices strategically selected measures under pay-for-performance was likely diminished by the uncertain payoff (ie, increase in bonuses or reduction in penalties) associated with selecting specific measures, since in the VM payment adjustments were based on a composite of performance measures assessed among all practices, and the set of measures and practices varied year-to-year.^16^ However, practices in the MIPS face similar uncertainty about the set of peers reporting on a given measure, and thus are also likely to rely on assessments of their own past performance relative to other practices when selecting measures.^46^ Second, findings from our difference-in-differences analyses may not generalize to Medicare beneficiaries living in institutional settings, such as nursing homes, because the fee-for-service Medicare CAHPS survey samples from the community-dwelling Medicare population. Third, our difference-in-differences analyses could have been biased by unmeasured confounders. However, we did not detect meaningful differential changes on observable patient or practice characteristics.

## Conclusions

In precursors of the MIPS, we found that large clinician practices were more likely to voluntarily include patient experience measures in a pay-for-performance program when they previously performed well on these measures. However, mandatory reporting of patient experiences was not associated with improved performance on these measures. These findings underscore concerns about gaming in the MIPS and provide cautionary evidence about proposed reforms to this program.

## Notes

Supplement. Section 1. Policy context eTable 1. Structure and phase-in of the VM and PQRS by year and clinician practice size Section 2. Fee-for-service Medicare CAHPS and CAHPS for PQRS surveys eTable 2. Items and domains in the CAHPS for PQRS survey and corresponding items in the Fee-for-service Medicare CAHPS survey eTable 3. Correlations of patient experience scores across domains in the CAHPS for PQRS survey eTable 4. Items with missing responses in the Fee-for-service Medicare CAHPS survey Section 3. Analysis of patient experience scores in the Fee-for-service Medicare CAHPS survey and concordance with practice scores from the CAHPS for PQRS survey Construction of domain and composite patient experience scores in the Fee-for-service Medicare CAHPS survey Concordance of patient experiences in the Fee-for-service Medicare CAHPS survey with practice-level scores from the CAHPS for PQRS survey eTable 5. Composite patient scores from the Fee-for-service Medicare CAHPS survey by quintile of practice scores Section 4. Measure selection and gaming analysis eTable 6. Characteristics of large practices and their patients, among large practices that reported CAHPS scores in 2016 and began reporting them in 2014 or 2015 eTable 7. Practice-level correlations of CAHPS scores across years Section 5. Difference-in-differences analysis Respondent sample for difference-in-differences analyses eFigure 1. Proportions of practices including CAHPS scores in the VM in 2014-2016 (N=140 practices that first reported CAHPS scores in 2014) eFigure 2. Sample inclusion criteria for difference-in-differences analysis Respondent characteristics eFigure 3. Means or proportions of patient characteristics in large vs. smaller practices from 2011-2013 and 2015-2016 eFigure 4. Proportions of large and smaller practices publicly reporting CAHPS measures in 2014 vs. 2015-2016 eFigure 5. Event-study plots of annual differential changes in composite and domain-level patient experience scores between large and smaller practices (relative to 2013) eTable 8. Preintervention practice characteristics and changes in the characteristics of large vs. smaller practices in difference-in-differences analysis eTable 9. Difference-in-differences estimates based on responses to concurrent Fee-for-service Medicare CAHPS surveys eTable 10. Difference-in-differences estimates among patients attributed to practices based on outpatient claims with primary care clinicians or specialists
