# Supplementary material for: Changes in Patient Experiences and Assessment of Gaming Among Large Clinician Practices in Precursors of the Merit-Based Incentive Payment System
Source: JAMA Health Forum. 2021 Oct 8;2(10):e213105. doi: 10.1001/jamahealthforum.2021.3105 (PMC8623747; doi:10.1001/jamahealthforum.2021.3105)
Supplement: Supplement. — Section 1. Policy context eTable 1. Structure and phase-in of the VM and PQRS by year and clinician practice size Section 2. Fee-for-service Medicare CAHPS and CAHPS for PQRS surveys eTable 2. Items and domains in the CAHPS for PQRS survey and corresponding items in the Fee-for-service Medicare CAHPS survey eTable 3. Correlations of patient experience scores across domains in the CAHPS for PQRS survey eTable 4. Items with missing responses in the Fee-for-service Medicare CAHPS survey Section 3. Analysis of patient experience scores in the Fee-for-service Medicare CAHPS survey and concordance with practice scores from the CAHPS for PQRS survey Construction of domain and composite patient experience scores in the Fee-for-service Medicare CAHPS survey Concordance of patient experiences in the Fee-for-service Medicare CAHPS survey with practice-level scores from the CAHPS for PQRS survey eTable 5. Composite patient scores from the Fee-for-service Medicare CAHPS survey by quintile of practice scores Section 4. Measure selection and gaming analysis eTable 6. Characteristics of large practices and their patients, among large practices that reported CAHPS scores in 2016 and began reporting them in 2014 or 2015 eTable 7. Practice-level correlations of CAHPS scores across years Section 5. Difference-in-differences analysis Respondent sample for difference-in-differences analyses eFigure 1. Proportions of practices including CAHPS scores in the VM in 2014-2016 (N=140 practices that first reported CAHPS scores in 2014) eFigure 2. Sample inclusion criteria for difference-in-differences analysis Respondent characteristics eFigure 3. Means or proportions of patient characteristics in large vs. smaller practices from 2011-2013 and 2015-2016 eFigure 4. Proportions of large and smaller practices publicly reporting CAHPS measures in 2014 vs. 2015-2016 eFigure 5. Event-study plots of annual differential changes in composite and domain-level patient experience scores between large and s [file jamahealthforum-e213105-s001.pdf]

Supplemental Online Content

Roberts ET, Song Z, Ding L, McWilliams JM. Changes in patient experiences and assessment of gaming among large clinician practices in precursors of the Merit-Based Incentive Payment System. *JAMA Health Forum*. 2021;2(10):e213105. doi:10.1001/jamahealthforum.2021.3105

Section 1. Policy context
eTable 1. Structure and phase-in of the VM and PQRS by year and clinician practice size
Section 2. Fee-for-service Medicare CAHPS and CAHPS for PQRS surveys **eTable 2.** Items and domains in the CAHPS for PQRS survey and corresponding items in the Fee-for-service Medicare CAHPS survey
eTable 3. Correlations of patient experience scores across domains in the CAHPS for PQRS survey
eTable 4. Items with missing responses in the Fee-for-service Medicare CAHPS survey
Section 3. Analysis of patient experience scores in the Fee-for-service Medicare CAHPS survey and concordance with practice scores from the CAHPS for PQRS survey
eTable 5. Composite patient scores from the Fee-for-service Medicare CAHPS survey by quintile of practice scores
Section 4. Measure selection and gaming analysis
eTable 6. Characteristics of large practices and their patients, among large practices that reported CAHPS scores in 2016 and began reporting them in 2014 or 2015
eTable 7. Practice-level correlations of CAHPS scores across years
Section 5. Difference-in-differences analysis
Respondent sample for difference-in-differences analyses
eFigure 1. Proportions of practices including CAHPS scores in the VM in 2014-2016 (N=140 practices that first reported CAHPS scores in 2014)
eFigure 2. Sample inclusion criteria for difference-in-differences analysis Respondent characteristics
eFigure 3. Means or proportions of patient characteristics in large vs. smaller practices from 2011-2013 and 2015-2016
eFigure 4. Proportions of large and smaller practices publicly reporting CAHPS measures in 2014 vs. 2015-2016

© 2021 Roberts ET et al. *JAMA Health Forum.*

eFigure 5. Event-study plots of annual differential changes in composite and domain-level patient experience scores between large and smaller practices (relative to 2013)
eTable 8. Pre-intervention practice characteristics and changes in the characteristics of large vs. smaller practices in difference-in-differences analysis
eTable 9. Difference-in-differences estimates based on responses to concurrent Fee-for-service Medicare CAHPS surveys
eTable 10. Difference-in-differences estimates among patients attributed to practices based on outpatient claims with primary care clinicians or specialists

This supplemental material has been provided by the authors to give readers additional information about their work.

© 2021 Roberts ET et al. *JAMA Health Forum.*

I. Policy context

We studied two precursors of Medicare's Merit-Based Incentive Payment System: the Value-Based Payment Modifier (VM), a pay-for-performance program, and the Physician Quality Reporting System (PQRS), a public reporting program. As detailed in **eTable 1,** the VM and PQRS were phased in over time, with different program components becoming mandatory based on the year and size of clinician practices.[1-9] We exploited the 2014 phase-in of a PQRS policy, which required large practices with ≥100 clinicians to publicly report patient experience measures from the Consumer Assessment of Healthcare Providers and Systems (CAHPS) survey, to study the association between mandatory public reporting and changes in performance on CAHPS measures. We also examined practices' decision to include CAHPS measures as an optional component of quality scores for the VM, whose pay-for-performance provisions were fully phased in for large practices by 2014. We analyzed annual practice-level scores on CAHPS measures from 2014-2016, when these scores were reported by CMS and practices could voluntarily include them in the VM. The MIPS replaced both the VM and PQRS in 2017 and made reporting CAHPS measures optional for all practices.

eTable 1: Structure and phase-in of the VM and PQRS by year and clinician practice size

	**Size of Clinician Practice:**		
**Performance Year:**	**Solo practices & groups with 2-9 clinicians**	**10-99 clinicians**	**≥100 clinicians**
**2011 and 2012**	• **PQRS:** Optional program with incentives for participation but no penalties for non-participation. Reporting patient experiences with care optional<br>• **VM:** not yet implemented		
**2013**	• **PQRS:** Mandatory program with penalties for non-participation, though reporting patient experiences was optional<br>• **VM:** did not apply		• **PQRS:** Mandatory program with penalties for non-participation, though reporting patient experiences was optional<br>• **VM:** Voluntary performance-based payment adjustments (practices exempt from penalties if they met PQRS reporting requirements)

© 2021 Roberts ET et al. *JAMA Health Forum.*

**2014** [b]	• **PQRS:** Mandatory program with penalties for non-participation, though reporting patient experiences was optional<br>• **VM:** did not apply	• **PQRS:** Mandatory program with penalties for non-participation, though reporting patient experiences was optional<br>• **VM:** Bonuses up to +2.0% x budget neutrality factor [a] based on overall quality and cost scores (practices exempt from penalties if they met PQRS reporting requirements)	• **PQRS:** Mandatory program with penalties for non-participation; reporting patient experiences with care was mandatory<br>• **VM:** Penalties or bonuses (-2.0% to up to +2.0% x budget neutrality factor [a]) based on overall quality and cost scores. Patient experiences were optional components of overall quality score
**2015**	• **PQRS:** Mandatory program with penalties for non-participation, though reporting patient experiences was optional<br>• **VM:** Bonuses up to +2.0% x budget neutrality factor [a] based on overall quality and cost scores (practices exempt from penalties if they met PQRS reporting requirements)	• **PQRS:** Mandatory program with penalties for non-participation, though reporting patient experiences was optional<br>• **VM:** Penalties or bonuses (-4.0% to up to +4.0% x budget neutrality factor [a]) based on overall quality and cost scores.	• **PQRS:** Mandatory program with penalties for non-participation; reporting patient experiences with care was mandatory<br>• **VM:** Penalties or bonuses (-4.0% to up to +4.0% x budget neutrality factor [a]) based on overall quality and cost scores. Patient experiences were optional components of overall quality score
**2016**	• **PQRS:** Mandatory program with penalties for non-participation, though reporting patient experiences was optional<br>• **VM:** Bonuses up to +2.0% x budget neutrality factor [a] based on overall quality and cost scores (practices exempt from penalties if they met PQRS reporting requirements)	• **PQRS:** Mandatory program with penalties for non-participation, though reporting patient experiences was optional<br>• **VM:** Bonuses up to +2.0% x budget neutrality factor [a] based on overall quality and cost scores (practices exempt from penalties if they met PQRS reporting requirements)	• **PQRS:** Mandatory program with penalties for non-participation; reporting patient experiences with care was mandatory<br>• **VM:** Bonuses up to +2.0% x budget neutrality factor [a] based on overall quality and cost scores (practices exempt from penalties if they met PQRS reporting requirements). Patient experiences were optional components of overall quality score

The table shows the phase-in of the programs by performance year; practices received payment adjustments related to these programs 2 years later.

[a] Budget neutrality factor was calculated by CMS and scaled percentage point payment adjustments so that VM bonuses and penalties were budget neutral in aggregate. Practices subject to the VM could receive additional upward payment adjustments for performance if they disproportionately served high-risk patients (mean Medicare HCC risk score of attributed patients in the upper quartile of Medicare beneficiaries nationally).

[b] Transitional year omitted from public reporting analyses.

© 2021 Roberts ET et al. *JAMA Health Forum.*

II. Fee-for-service Medicare CAHPS and CAHPS for PQRS surveys

We analyzed data from two main sources. First, we analyzed practice-level data from VM Practice Files. These files included annual patient experience scores for practices that reported these measures for the PQRS. The Centers for Medicare and Medicaid Services (CMS) calculated these scores based on responses to the CAHPS for PQRS survey, which was administered annually to a random sample of fee-for-service Medicare beneficiaries in practices where beneficiaries received most of their primary care.[10] Only practices that reported patient experiences with care for the PQRS were included in the survey. CMS reported practice-level scores for 11 patient experience domains assessed in the CAHPS for PQRS survey **(eTable 2)**.

Practice-level scores were publicly reported by CMS,[11,12] though practices could voluntarily include them in an overall quality score for the VM.[2,13] (Practices could include all or none of the 11 CAHPS domain scores in their overall VM quality score.[14]) The overall quality score was one of two factors that determined whether practices received performance-based payment adjustments—i.e., bonuses or penalties—through the VM (the other factor was per-patient spending).[1-6] We analyzed annual scores for large practices (≥100 clinicians) from 2014-2016, when these scores were publicly reported and practices could voluntarily include them in the VM.

Second, we analyzed patient-level data from the Fee-for-service Medicare CAHPS survey, which is separate from, but closely related to, the CAHPS for PQRS survey.[13] The Fee-for-service Medicare CAHPS survey was administered annually to a representative sample of individuals enrolled in traditional (i.e., fee-for-service) Medicare and who lived in the community.[15] We analyzed this survey to assess changes in patient experiences with care associated with the 2014 phase-in of mandated public reporting on CAHPS patient experience measures.[2,10,16] Specifically, we analyzed changes in patient experiences from 2011-2013 (prior to the public reporting mandate) to 2015-2016 (2-3 years following the mandate's introduction)

© 2021 Roberts ET et al. *JAMA Health Forum.*

using a difference-in-differences analysis and omitted 2014 as a transitional year. Consistent with prior research,[17,18] we used surveys administered in year *t* (i.e., 2012-2014 and 2016-2017) to measure patient experiences with care in year *t-1* (i.e., 2011-2013 and 2015-2016), since the surveys were conducted early in the year and asked respondents to rate their experiences with care over the prior 6 months.

This section identifies survey items and domains common to both the Fee-for-service Medicare CAHPS and CAHPS for PQRS surveys and provides descriptive statistics of practice-level patient experience scores reported by CMS (based on the CAHPS for PQRS survey). **eTable 2** displays domains of patient experiences assessed in the CAHPS for PQRS survey and corresponding items and domains in the Fee-for-service Medicare CAHPS survey.[13] Five of the 11 domains in the CAHPS for PQRS survey had closely corresponding items in the Fee-for-service Medicare CAHPS surveys from 2012-2014 and 2016-2017: rating of primary physician, physician communication, timely access to care, access to specialists, and care coordination. Therefore, we focused on performance in these 5 domains in analyses of mandatory public reporting, which used the Fee-for-service Medicare CAHPS survey.

To examine whether these 5 domains were representative of performance across all domains assessed in the CAHPS for PQRS survey, we computed correlations of practice-level scores among all 11 domains in the CAHPS for PQRS survey. Domain scores were strongly correlated within practices (ρ>0.5 for 10 domain pairs and ρ>0.2 for 34 domain pairs, out of 55 total pairs; **eTable 3**), including between the 5 domains included in our analyses and the 6 excluded domains (ρ>0.2 for 19 out of 30 domain pairs). Because measurement error can substantially attenuate estimates of correlations in small samples (such as those collected for practices in the CAHPS for PQRS survey),[19] the true correlations may be higher.

Finally, we examined the proportion of survey items with missing data in the 5 patient experience domains we analyzed from the Fee-for-service Medicare CAHPS survey. Missing

© 2021 Roberts ET et al. *JAMA Health Forum.*

data can be a result of item non-response, skip patterns, or the exclusion of items in certain survey years. We found that missing response rates were comparable in large vs. smaller practices, and except for 1 item (seeing a physician within 15 minutes of appointment time), did not change differentially between these two groups of practices between the 2012-2014 and 2016-2017 surveys **(eTable 4)**. These findings support our use of these data in a difference-in-differences design, since the proportions of items with non-missing responses contributing to the analyses did not change differentially between large and smaller practices over time.

© 2021 Roberts ET et al. *JAMA Health Forum.*

eTable 2: Items and domains in the CAHPS for PQRS survey and corresponding items in the Fee-for-service Medicare CAHPS survey

		**Corresponding items in Fee-for-service Medicare CAHPS survey:**				
		**Survey year:** [a]				
**CAHPS for PQRS survey Items and domains**	**Item Scale** [b]	**2012**	**2013**	**2014**	**2016**	**2017**
**Rating of physician domain**						
Rating of primary physician	0-10	Yes	Yes	Yes	Yes	Yes
**Physician communication domain**						
Physician explained things in a way that was easy to understand	1-4	Yes	Yes	Yes	Yes	Yes
Physician listened carefully to you	1-4	Yes	Yes	Yes	Yes	Yes
Physician gave you easy to understand instructions about health problem or concern	N/A	No	No	No	No	No
Physician knew important information about your medical history	N/A	No	No	No	No	No
Physician showed respect for what you had to say	1-4	Yes	Yes	Yes	Yes	Yes
Physician spent enough time with you	1-4	Yes	Yes	Yes	Yes	Yes
**Timely access to care domain**						
Got urgent care appointment as soon as you needed	1-4	Yes	Yes	Yes	Yes	Yes
Got appointment for check-up or routine care as soon as you wanted	1-4	Yes	Yes	Yes	Yes	Yes
Called physician's office during regular hours and got answer to medial questions same day	N/A	No	No	No	No	No
Called physician's office after hours and got answers to medical questions as soon as you needed	1-4	No	Yes	Yes	Yes	No
Saw physician within 15 minutes of appointment time	1-4	Yes	Yes	Yes	Yes	Yes
Ease of getting care, tests, or treatment you thought you needed	1-4	Yes	Yes	Yes	Yes	Yes

© 2021 Roberts ET et al. *JAMA Health Forum.*

**Access to specialists domain [c]**						
Ease of making appointments with specialists	1-4	Yes	Yes	Yes	Yes	Yes
Specialist who you saw most often knew the important information about your medical history	N/A	No	No	No	No	No
**Care coordination domain**						
Physician had your medical records during your visits	1-4	Yes	Yes	Yes	Yes	Yes
Physician's office followed up to give you the results of a text or x-ray	1-4	Yes	Yes	Yes	Yes	Yes
You got help from your care team to manage care, tests, or treatment from different physicians	1-3	Yes	Yes	Yes	Yes	Yes
Satisfaction with help from your care team to manage care, tests, or treatment from different physicians	N/A	No	No	No	No	No
Primary doctor informed about care provided by specialists [d]	1-4	Yes	Yes	Yes	Yes	Yes
**Medication review domain [e]**						
Health care physician reviewed your medications and answered questions	Yes/No	No	No	Yes	Yes	Yes
Review increased your understanding of how to take medications	1-3	No	No	Yes	Yes	Yes
Physician gave written information about how to take prescription medicines that was easy to understand [f]	1-3	No	No	Yes	Yes	Yes
Physician suggested ways to help you remember to take your medicines	N/A	No	No	No	No	No
**Communication between visits domain [e]**						
Got reminders from physician's office between visits	Yes/No	No	Yes	Yes	Yes	No
Got reminder from physician's office to make an appointment for tests or treatment	Yes/No	No	Yes	Yes	Yes	No
**Shared decision-making domain [e]**	N/A	No	No	No	No	No
**Health promotion and education domain [e]**	N/A	No	No	No	No	No
**Stewardship of patient resources domain [e]**	N/A	No	No	No	No	No

© 2021 Roberts ET et al. *JAMA Health Forum.*

**Office staff courtesy domain [e]**	N/A	No	No	No	No	No

[a] We omitted 2015 survey data pertaining to the 2014 transition year.
[b] Numeric scores range from 0 (worst) to 10 (best); from 1 (never) to 4 (always); or have values of 1 (no), 2 (yes, somewhat), or 3 (yes, definitely). Prior to analysis, numeric scores were converted to a consistent 0-100 scale. For items part of domain scores, we first subtracted the overall mean for the item and calculated a domain score at the patient level as an equally weighted average of items with non-missing responses.
[c] The access to specialists domain in the CAHPS for PQRS survey included an item about the number of specialists a patient saw. Because this item does not directly assess quality of care, we did not include it when constructing the "access to specialists" domain score from the Fee-for-service Medicare CAHPS survey.
[d] We included this item to construct a care coordination domain score in analyses using the Fee-for-service Medicare CAHPS survey, although this item was not included in the CAHPS for PQRS survey.
[e] Domain not included in our analyses of patient-level survey data because items were not assessed in the Fee-for-service Medicare CAHPS survey in all study years.
[f] Shown here is the item from the Fee-for-service Medicare CAHPS survey, which corresponded to two items in the CAHPS for PQRS survey: (1) Physician gave easy-to-understand instructions about how to take prescription medicines; (2) Physician gave information in writing about how to take prescription medicines that was easy to understand.

© 2021 Roberts ET et al. *JAMA Health Forum.*

eTable 3: Correlations of patient experience scores across domains in the CAHPS for PQRS survey

**Domain of patient experience**	Rating of primary physician	Physician communication	Timely access to care	Access to specialists	Care coordination	Medication review	Communication between visits	Shared decision-making	Health promotion and education	Stewardship of patient resources	Office staff courtesy
Rating of primary physician [a]	1.000	0.928	0.571	0.261	0.763	0.371	-0.079	0.468	0.229	0.258	0.716
Physician communication [a]	0.928	1.000	0.581	0.277	0.800	0.348	-0.077	0.472	0.209	0.249	0.747
Timely access to care [a]	0.571	0.581	1.000	0.381	0.611	0.178	0.027	0.362	0.167	0.153	0.631
Access to specialists [a]	0.261	0.277	0.381	1.000	0.373	0.161	0.078	0.065	0.211	0.192	0.249
Care coordination [a]	0.763	0.800	0.611	0.373	1.000	0.259	0.079	0.438	0.269	0.259	0.721
Medication review	0.371	0.348	0.178	0.161	0.259	1.000	0.213	0.238	0.336	0.114	0.185
Communication between visits	-0.079	-0.077	0.027	0.078	0.079	0.213	1.000	0.013	0.155	-0.086	-0.021
Shared decision-making	0.468	0.472	0.362	0.065	0.438	0.238	0.013	1.000	0.261	0.190	0.391
Health promotion and education	0.229	0.209	0.167	0.211	0.269	0.336	0.155	0.261	1.000	0.142	0.100

© 2021 Roberts ET et al. *JAMA Health Forum.*

Stewardship of patient resources	0.258	0.249	0.153	0.192	0.259	0.114	-0.086	0.190	0.142	1.000	0.131
Office staff courtesy	0.716	0.747	0.631	0.249	0.721	0.185	-0.021	0.391	0.100	0.131	1.000

This table displays correlations of practice-level domain scores based on the CAHPS for PQRS survey. We analyzed risk-adjusted, standardized scores reported by CMS in the VM Practice File. The unit of analysis was the practice-year (N=742 practice-years). We included 301 practices that reported patient experiences in each year from 2014-2016 (N=140 practices) or in 2015 and 2016 (N=161 practices).
[a] Domains included in our analyses of the Fee-for-service Medicare CAHPS surveys.

© 2021 Roberts ET et al. *JAMA Health Forum.*

eTable 4: Items with missing responses in the Fee-for-service Medicare CAHPS survey

Items in 5 Fee-for-service Medicare CAHPS survey domains that corresponded to items in the CAHPS for PQRS survey

	Survey respondents attributed to practices with [b]				
	50-89 clinicians		111-150 clinicians		
**Proportion of items with missing responses [a]**	Surveys administered from 2012-2014	Surveys administered from 2016-2017	Surveys administered from 2012-2014	Surveys administered from 2016-2017	**P-value [c]**
**Rating of physician domain**					
Rating of primary physician	15.9%	17.2%	16.1%	16.0%	0.21
**Physician communication domain**					
Physician explained things in a way that was easy to understand	15.8%	16.2%	16.0%	15.1%	0.18
Physician listened carefully to you	15.8%	16.8%	16.0%	15.8%	0.24
Physician showed respect for what you had to say	15.8%	16.8%	15.8%	15.8%	0.34
Physician spent enough time with you	15.8%	16.9%	16.0%	15.7%	0.17
**Timely access to care domain**					
Got urgent care appointment as soon as you needed	63.0%	59.5%	63.2%	60.1%	0.81
Got appointment for check-up or routine care as soon as you wanted	16.4%	14.5%	16.7%	13.1%	0.10
Called physician's office after hours and got answers to medical questions as soon as you needed	93.9%	95.0%	94.0%	94.8%	0.81
Saw physician within 15 minutes of appointment time	12.4%	11.3%	13.8%	10.0%	0.01
Ease of getting care, tests, or treatment you thought you needed	39.0%	19.6%	39.6%	19.8%	0.71

© 2021 Roberts ET et al. *JAMA Health Forum.*

**Access to specialists domain**					
Ease of making appointments with specialists	46.0%	38.5%	47.0%	38.6%	0.57
**Care coordination domain**					
Physician had your medical records during your visits	16.1%	17.1%	16.3%	15.9%	0.21
Physician's office followed up to give you the results of a text or x-ray	26.6%	28.0%	25.9%	27.1%	0.86
You got help from your care team to manage care, tests, or treatment from different physicians	82.5%	80.5%	81.9%	79.2%	0.54
Primary doctor informed about care provided by specialists [d]	46.4%	41.3%	46.3%	41.1%	0.91

[a] In patient-level analyses, we analyzed items from five domains of the Fee-for-service Medicare CAHPS survey that corresponded to domains in the CAHPS for PQRS survey. Because the Fee-for-service Medicare CAHPS survey is administered early in the year and asks patients to rate their experiences with care over the previous six months, we analyzed surveys administered from 2012-2014 and 2016-2017 to assess patient experiences with care in 2011-2013 and 2015-2016, respectively. The 2015 survey, pertaining to 2014 outcomes, is omitted. Shown are the proportions of survey items with missing responses, among respondents to the 2012-2014 or 2016-2017 surveys attributed to practices with 50-89 vs. 111-150 clinicians.
[b] We attributed each Medicare beneficiary to the practice that accounted for the plurality of the beneficiary’s office visits with primary care clinicians in the year prior to the survey.
[c] P-value for the differential change in item-level missingness from the 2012-2014 to the 2016-2017 Fee-for-service Medicare CAHPS surveys, among survey respondents attributed to practices with 50-89 vs. 111-150 clinicians. Differential changes estimated from a respondent-level linear regression model, which estimated missingness in each item as a function of practice size (111-150 clinicians, with 50-89 clinicians as the reference), survey period (2016-2017, with 2012-2014 as the reference), and an interaction between practice size and survey period. P-values calculated from standard errors clustered at the practice (taxpayer identification number) level. We did not adjust for survey weights or respondent-level characteristics.
[d] We included this item to construct a care coordination domain score in analyses using the Fee-for-service Medicare CAHPS survey, although this item was not included in the CAHPS for PQRS survey.

© 2021 Roberts ET et al. *JAMA Health Forum.*

III. Analysis of patient experience scores in the Fee-for-service Medicare CAHPS survey and concordance with practice scores from the CAHPS for PQRS survey

This section describes how we constructed domain-level and composite patient experience scores in patient-level analyses of the Fee-for-service Medicare CAHPS survey and how these compare to scores that were publicly reported for practices based on the CAHPS for PQRS survey.

Construction of domain and composite patient experience scores in the Fee-for-service Medicare CAHPS survey

We aggregated items from the Fee-for-service Medicare CAHPS survey into patient-level scores for 5 patient experience domains that closely corresponded to domains in the CAHPS for PQRS survey **(eTable 2).** To construct these domain scores, we first subtracted the overall mean for each item, used a linear transformation to convert all items to a consistent 0-100 scale, and calculated a patient-level domain score as an equally weighted average of the domain's constituent items. (Items with missing responses were excluded from domain scores.) We also constructed a composite patient-level score, which we defined as an equally weighted average of the 5 domain scores. Since item-level missingness was comparable over time between large and smaller practices in our difference-in-differences analysis **(eTable 4)**, our approach to constructing domain scores should not assign systematically different weights to items for large vs. smaller practices across different periods.

Concordance of patient experiences in the Fee-for-service Medicare CAHPS survey with practice-level scores from the CAHPS for PQRS survey

The CAHPS for PQRS survey is separate from, but closely related to, the Fee-for-service Medicare CAHPS survey. Given the similarity of the surveys **(eTable 2),** we expect that practice-level scores based on the CAHPS for PQRS survey will be positively correlated with the mean scores of patients in those practices who were sampled in the Fee-for-service Medicare CAHPS survey. To examine the concordance of scores in the two surveys, we first

© 2021 Roberts ET et al. *JAMA Health Forum.*

grouped practices into quintiles of their average patient experience score in 2016 (equally weighted average of scores for 11 patient experience domains reported by CMS). We then compared mean composite patient-level scores from the 2017 Fee-for-service Medicare CAHPS survey across quintiles of practices. (We analyzed the 2017 Fee-for-service Medicare CAHPS survey to assess patient experiences in 2016.) We found that composite patient-level scores were higher in practices with higher scores—e.g., 78.8 points in the lowest quintile and 82.5 points in the highest quintile of practices (P=0.01 for the difference; **eTable 5**). This is what we would expect, as the CAHPS for PQRS survey is administered to a representative sample of a practice's Medicare patients.

eTable 5: Composite patient scores from the Fee-for-service Medicare CAHPS survey by quintile of practice scores

	**Composite patient experience score** [b]	
**Quintile of practice-level patient experience score** [a]	**Mean**	**95% CI**
1 (Lowest quintile – lowest overall rating of patient experiences)	78.8	(76.1, 80.2)
2	81.8	(80.4, 83.8)
3	81.4	(79.3, 83.0)
4	82.5	(80.5, 83.5)
5 (Highest quintile – highest overall rating of patient experiences)	82.5	(81.8, 84.4)

[a] We grouped practices into quintiles of their average patient experience score in 2016, which we calculated as an equally weighted average of scores from 11 patient experience domains reported by CMS in the VM Practice File (based on the CAHPS for PQRS survey). We analyzed risk-adjusted, standardized practice scores reported by CMS in the VM Practice File.
[b] Composite patient experience score, assessed from the 2017 Fee-for-service Medicare CAHPS survey. Means and 95% confidence intervals are from a linear regression that estimated patient-level composite scores as a function of practice quintiles. Analyses based on 256 practices and 6,511 respondents to the 2017 Fee-for-service Medicare CAHPS survey who were attributed to these practices based on 2016 primary care claims.

© 2021 Roberts ET et al. *JAMA Health Forum.*

IV. Measure selection and gaming analysis

In our first set of analyses, we examined the relationship between large practices' initial scores on CAHPS measures and subsequent inclusion of these measures in the VM. We focused on practices that reported CAHPS measures in 2016 and began reporting them in either 2014 or 2015, as this allowed us to assess how practice decisions to include CAHPS measures in the VM changed as they learned about their prior performance. We excluded practices that participated as an ACO in the Medicare Shared Savings Program (MSSP) in any year from 2012-2016 as these practices were not initially included in the VM and reported patient experience measures through a separate CAHPS for ACOs survey.[2,20] Our analysis sample consisted of 301 practices: 140 that that began reporting CAHPS measures in 2014 and 161 that began reporting these measures in 2015.

eTable 6 presents selected characteristics of these practices. We note that the figures below reflect characteristics of practices' patient populations with fee-for-service Medicare, but not necessarily the characteristics of respondents to the CAHPS for PQRS surveys, since we only had access to practice-level CAHPS scores for this analysis.

eTable 6: Characteristics of large practices and their patients, among large practices that reported CAHPS scores in 2016 and began reporting them in 2014 or 2015

**Characteristic of practice in baseline year** [a]	**Mean or proportion (25th percentile, 75th percentile)**
Number of clinicians billing under practice taxpayer identification number (TIN)	431 (157, 498)
Number of fee-for-service Medicare beneficiaries attributed to practice TIN	10,229 (4,435, 11,337)
Age of attributed beneficiaries (years)	71.6 (70.4, 73.2)
Proportion of beneficiaries who are female (%)	55.8 (54.3, 57.7)
Proportion of beneficiaries with full Medicaid (%)	17.8 (10.2, 22.2)
Proportion of beneficiaries with partial Medicaid (%) [b]	5.0 (2.0, 6.8)
Proportion of beneficiaries who are disabled (%) [c]	26.7 (20.7, 31.0)
HCC risk score of attributed beneficiaries	1.4 (1.2, 1.5)

[a] Practice-level characteristic, shown for the first year in which practices publicly reported CAHPS scores. The unit of analysis is the practice taxpayer identification number. The sample consists of 301 practices.
[b] That is, enrollment in one of the Medicare Savings Programs, which provide partial Medicaid benefits that pay for Medicare Part B premiums and in some cases, Parts A and B cost sharing for beneficiaries meeting income and asset eligibility criteria.

© 2021 Roberts ET et al. *JAMA Health Forum.*

[b] That is, disability was the beneficiary's original reason for Medicare entitlement.

Practices were scored annually based on their patients' responses to the CAHPS for PQRS survey. In each year, practices could choose to include these measures in an overall quality score for the VM. Since practices received scores on CAHPS measures after they would have decided to include them in the VM, information about prior performance would have guided practices' selection of these measures for the VM. As shown in **eTable 7,** CAHPS scores were highly correlated within practices across years (e.g., $\rho>0.6$ for 8 of 11 domains assessed in 2014 and 2016). These findings demonstrate that prior scores were a reliable signal of future performance and motivate our analyses of practice decisions to include CAHPS measures in the VM as a function of their baseline performance on these measures.

eTable 7: Practice-level correlations of CAHPS scores across years

	**Correlation between years**		
	**2014 and 2016**	**2014 and 2015**	**2015 and 2016**
**Domain of patient experience** [a]	n=140 TINs	n=140 TINs	n=301 TINs
Rating of primary physician	0.655	0.573	0.818
Physician communication	0.648	0.527	0.857
Timely access to care	0.634	0.705	0.615
Access to specialists	0.552	0.545	0.383
Care coordination	0.700	0.726	0.837
Medication review	0.332	0.415	0.426
Communication between visits	0.708	0.743	0.674
Shared decision-making	0.547	0.492	0.572
Health promotion and education	0.823	0.790	0.808
Stewardship of patient resources	0.653	0.668	0.697
Office staff courtesy	0.676	0.709	0.881

[a] Correlations of patient experience scores reported at the practice level by domain from the CAHPS for PQRS survey (table rows) across years (table columns). We analyzed risk-adjusted, standardized scores reported by CMS in the VM Practice File. We included 301 practices that publicly reported patient experiences in each year from 2014-2016 (N=140 practices) or in 2015 and 2016 (N=161 practices).

We measured each practice's baseline performance as an equally weighted average of scores on 11 patient experience domains in the first year the practice reported CAHPS measures for the PQRS (either 2014 or 2015 in our sample). We grouped practices into quintiles of their baseline performance and compared the proportion of practices that included CAHPS measures in the VM, at baseline and 1-2 years later, across quintiles of practices'

© 2021 Roberts ET et al. *JAMA Health Forum.*

baseline scores. (Scores for subsequent years were based on patient-reported experiences with care in those years.)

We compared the proportions of practices that voluntarily included CAHPS scores in the VM at baseline and up to 2 years later across quintiles of practices' baseline scores. To do so, we fitted practice-level linear probability models of the form:

$$Elect\_Year\_t_k = \alpha + \sum_{q=2}^{5} \beta_q \left(Quintile_{k,t=0} = q\right) + \lambda 2015\_\text{Cohort}_k + \varepsilon_{kt} \tag{1}$$

where $Elect\_Year\_t_k$ indicates that a practice elected to include CAHPS scores for year *t* in its overall VM performance score for year *t*; $Quintile_{k,t=0} = q$ are indicators of the practice's baseline quintile (quintile 1, which includes the 20% of practices with the lowest baseline performance, is omitted as the reference); and 2015_Cohort_k indicates that a practice first reported CAHPS measures for the PQRS in 2015 (vs. 2014). We estimated separate models for election in the baseline year, 1 year after baseline, and 2 years after baseline. (Because the final model is estimated only on the 2014 cohort, which we follow through 2016, the cohort indicator falls out of this model.) Thus, estimates of β_q represent differences in the probability that practices in quintiles 2-5 vs. 1 at baseline elected to include their year *t* CAHPS scores in the VM (pooled across the 2015 and 2014 cohorts), adjusted for the mean difference in the proportion of practices from the 2015 and 2014 cohorts electing to include CAHPS scores in the VM. Figure 1 of the main manuscript graphs these estimates in *levels*, calculated as $\hat{\alpha}$ for quintile 1 and $\hat{\alpha} + \hat{\beta}_q$ for quintiles 2-5.

As shown in Figure 1, in the baseline year, 65.9% of practices in the lowest quintile of baseline performance elected to include CAHPS scores (measured at baseline) in the VM, compared with 78.9% of practices in the highest quintile of baseline performance (P=0.13 for the difference). One year after baseline, 66.1% of practices in the lowest baseline quintile vs. 77.4% of practices in the highest baseline quintile included CAHPS scores (measured one year

© 2021 Roberts ET et al. *JAMA Health Forum.*

after baseline) in the VM (P=0.18 for the difference). Two years after baseline, 67.9% in the lowest baseline quintile vs. 96.3% of practices in the highest baseline quintile included CAHPS scores in the VM (P=0.004 for the difference). Since practices were informed of their scores after CAHPS measures could have contributed to annual quality scoring for the VM, these findings suggest that practice decisions to include CAHPS measures in the VM were likely influenced by knowledge of performance revealed by prior scores.

To further examine patterns of measure selection among practices that reported CAHPS measures in multiple years, we focused on the cohort of 140 practices that first reported these measures in 2014 and again reported them in 2015 and 2016. We compared the proportions of practices that included CAHPS measures in the VM in from 2014-2016 across quintiles of practices' baseline performance scores. Results are plotted in **eFigure 1.** We also fitted a practice-level linear model to compare the proportions of practices in the lowest vs. higher quintiles of baseline performance that included CAHPS scores in the VM across years:

$$Elect_{kt} = \alpha + \sum_{q=2}^{5} \beta_q \left(Quintile_{k,t=0} = q\right) + \varphi 2015_t + \lambda 2016_t + \sum_{q=2}^{5} \theta_q \left(Quintile_{k,t=0} = q\right) \times 2015_t + \sum_{q=2}^{5} \delta_q \left(Quintile_{k,t=0} = q\right) \times 2016_t + \varepsilon_{kt} \quad (2)$$

Above, $Elect_{kt}$ indicates whether practice *k* elected to include CAHPS measures in the VM in year *t;* $\left(Quintile_{k,t=0} = q\right)$ are indicators for the quintile of the practice's baseline performance (the bottom quintile is omitted as the reference); and 2015_t and 2016_t are indicators for the 2015 and 2016 performance years, respectively. We tested the statistical significance of coefficients θ_5 and δ_5 on the interactions between indicator variables for the top baseline quintile and performance year. In this model, θ_5 represents the differential change in the proportion of practices in the highest vs. lowest quintiles of baseline performance that included CAHPS scores in the VM in 2015 vs. 2014; δ_5 is the analogous differential change in 2016 vs. 2014.

© 2021 Roberts ET et al. *JAMA Health Forum.*

As shown in **eFigure 1,** from 2014-2015, the proportion of practices in the lowest baseline quintile that included CAHPS measures in the VM declined by 14.3 percentage points, while the proportion of practices in the highest baseline quintile including these measures increased by 7.4 percentage points (differential change given by θ_5= 21.7 percentage points; P=0.20). From 2014-2016, the proportion of practices in the lowest baseline quintile that included CAHPS measures in the VM declined by 17.9 percentage points while the proportion in the highest baseline quintile increased by 29.6 percentage points (differential change given by δ_5= 47.5 percentage points; P=0.002). These findings are consistent with selective reporting—i.e., practices with higher initial scores becoming more likely to include them in the VM, and practices with lower initial scores becoming less likely, over time.

eFigure 1: Proportions of practices including CAHPS scores in the VM in 2014-2016 (N=140 practices that first reported CAHPS scores in 2014)

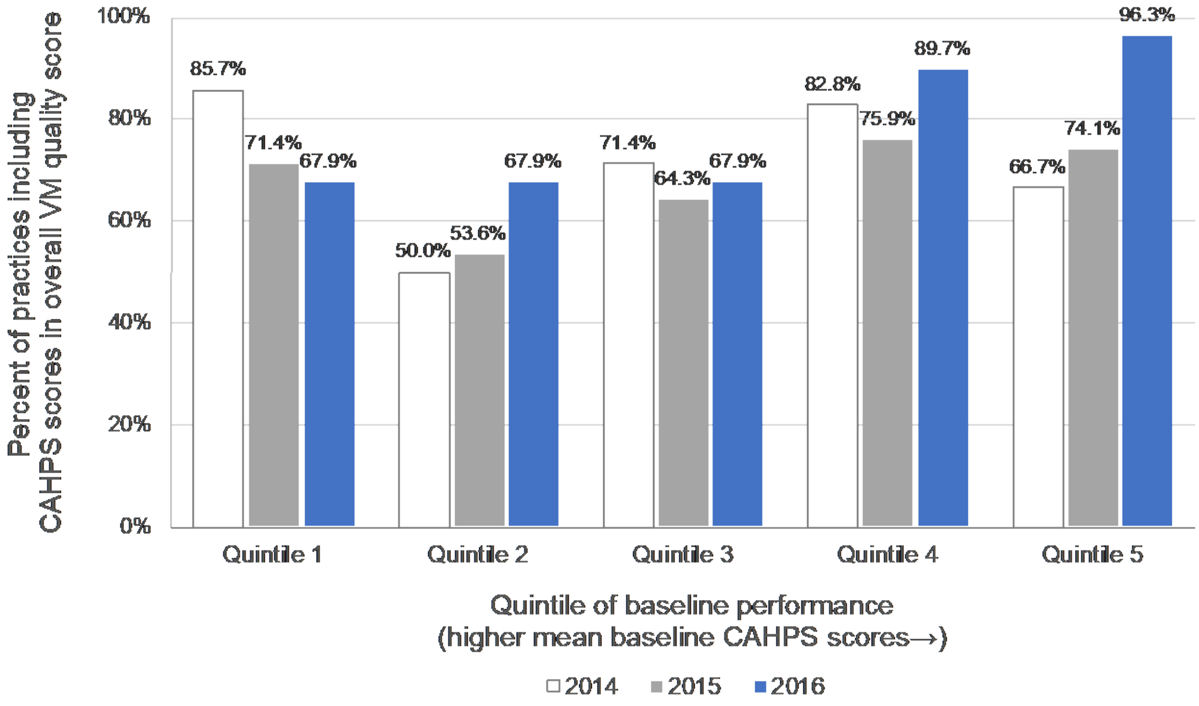

Estimates based on 140 large practices that initially reported CAHPS measures in 2014 and also reported them in 2015 and 2016. We measured each practice's baseline performance as an equally weighted average of scores on the 11 patient experience domains in 2014. Practice-level scores in these 11 domains were reported by CMS in the VM Practice File.

© 2021 Roberts ET et al. *JAMA Health Forum.*

V. Difference-in-differences analysis

Next, we used a difference-in-differences design to evaluate whether the phase-in of a PQRS policy that made public reporting of CAHPS measures mandatory for large practices (≥100 clinicians) was associated with differential improvements in patient experiences with care. This section describes the study sample used in this analysis; provides additional details about the difference-in-differences design, interpretation of our estimates, and tests of this study design's assumptions; and presents the results of sensitivity analyses.

Respondent sample for difference-in-differences analyses

We conducted difference-indifferences analyses using patient-level survey data from Fee-for-service Medicare CAHPS surveys. We analyzed surveys administered from 2012-2014 and 2016-2017 to assess patient experiences with care from 2011-2013 (before the public reporting mandate) and 2015-2016 (following the mandate's introduction). We omitted the 2015 survey, pertaining to patient experiences in 2014, as a transitional year. Consistent with prior research,[17,18] we attributed each survey respondent to one practice (identified by the taxpayer identification number [TIN]) that accounted for the majority of the respondent's outpatient primary care visits in the year preceding the survey year (e.g., in 2011 for respondents to the 2012 survey). Attributing patients in the year prior to the survey aligns with the period for which we assessed patient experiences with care. We excluded respondents who did not have any primary care claims needed for attribution to practices. We attributed respondents to practices based on primary care visits because key items in the CAHPS survey focus on primary care (e.g., ratings of primary physicians). We conducted sensitivity analyses (below) among patients attributed to practices based on outpatient visits with primary care clinicians or specialists.

We excluded respondents attributed to practices participating in a Medicare Shared Savings Program (MSSP) ACO in any year from 2012-2016, because ACO practices of all sizes were required to report patient experiences through a separate CAHPS for ACOs survey.[13] To

© 2021 Roberts ET et al. *JAMA Health Forum.*

avoid bias from compositional changes in the set of practices analyzed across study years, we excluded practices that participated in an MSSP ACO at any point from 2012-2016 from *all* study years, rather than only years in which practices participated in ACOs. Excluding practices only in years in which they participated in MSSP ACOs could bias our difference-in-differences estimates if, for example, practices entering ACOs differed in size and quality of care from practices not entering ACOs.

Lastly, we limited our analyses to respondents in practices with 50-89 or 111-150 clinicians. We excluded practices with 90-110 clinicians to mitigate attenuation bias from small year-to-year fluctuations in practice size that could have affected practice exposure to the reporting mandate. Our final sample consisted of 21,738 Medicare beneficiaries and 2,186 practice-years. **eFigure 2** depicts the derivation of this sample.

© 2021 Roberts ET et al. *JAMA Health Forum.*

eFigure 2: Sample inclusion criteria for difference-in-differences analysis

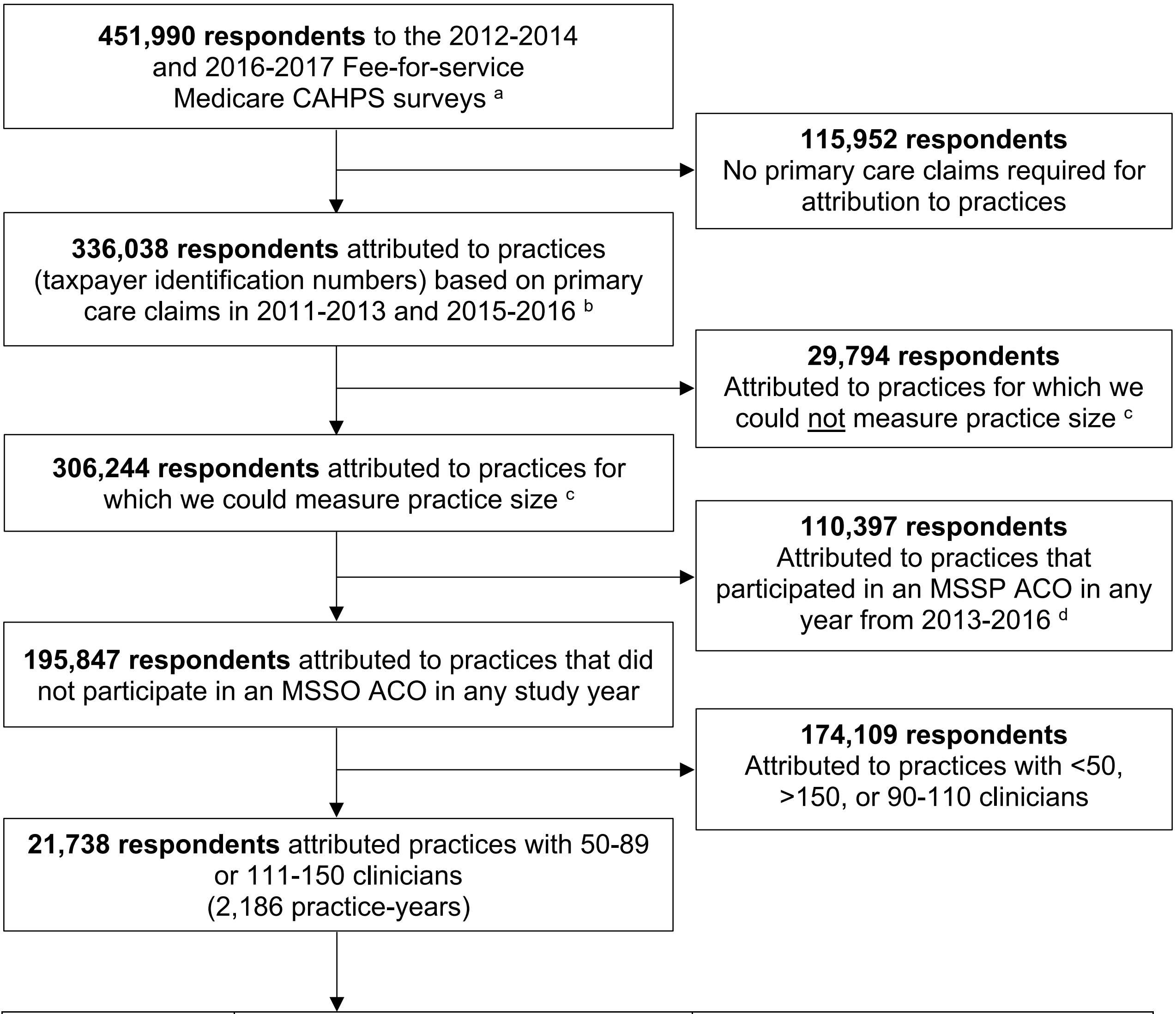

**Survey years:** [e]	**Practices with 50-89 clinicians:**	**Practices with 111-150 clinicians:**
2012-14 surveys	9,399 respondents, 945 practice-years	4,351 respondents, 344 practice-years
2016-17 surveys	4,999 respondents, 635 practice-years	2,989 respondents, 262 practice-years

[a] The Fee-for-service Medicare CAHPS survey is administered early in the year and asks respondents to rate their experiences with care over the prior 6 months. Thus, we analyzed the 2012-2014 and 2016-2017 surveys to assess experiences with care in 2011-2013 and 2015-2016. We omitted the 2015 survey, pertaining to patient experiences in 2014, as a transitional year.
[b] Respondents attributed to practices where they received the majority of primary care visits in the year prior to the survey (to align with the period in which we measure patient experiences with care). Practices were identifier using taxpayer identification numbers in Medicare claims.
[c] Practice size measured annually from MD-PPAS files as the number of unique clinicians billing under a practice taxpayer identification number (TIN).
[d] Because the MSSP ACO program began in 2012, we excluded practices in 2011 that subsequently participated in an MSSP ACO.
[e] 2012-14 survey years used to assess patient experiences with care in 2011-2013; 2016-17 survey years used to assess patient experiences with care in 2015-16.

© 2021 Roberts ET et al. *JAMA Health Forum.*

Practice size

In analyses using the Fee-for-service Medicare CAHPS survey (conducted among large and smaller practices), we measured practice size annually as the number of unique clinicians that billed under a practice taxpayer identification number (TIN), which we assessed from clinician-TIN billing relationships captured in Medicare Provider Practice and Specialty (MD-PPAS) files.[21] We measured practice size in the year prior to the CAHPS survey year to align this variable with the period for which we assessed patient experiences with care.

Respondent characteristics

We controlled for respondent characteristics using data from the following sources:

**Characteristic**:	**Data source and notes**:
▪ Age	▪ Medicare beneficiary summary file (MBSF).
▪ Sex	▪ MBSF.
▪ Race/ethnicity	▪ RTI race variable in MBSF.[22]
▪ Disability	▪ "OREC" variable in MBSF indicating that disability (SSDI) was the beneficiary's original reason for Medicare entitlement.
▪ End-stage renal disease	▪ "CREC" variable in MBSF indicating that end-stage renal disease is the beneficiary's current reason for Medicare entitlement.
▪ CMS HCC Risk score	▪ Medicare inpatient and outpatient claims 2 years prior to the CAHPS survey year and demographic characteristics (age, sex, disability, Medicaid enrollment) in the year prior to the CAHPS survey.[23]
▪ Count of CCW chronic conditions	▪ MBSF, chronic conditions segment (indicators of 27 chronic conditions reported on Medicare claims prior to the survey year).
▪ Enrollment in Medicaid	▪ Enrollment in full Medicaid for ≥1 month of the year, based on monthly dual eligibility enrollment codes in the MBSF. We used the presence of dual eligibility codes '02', '04' and '08' to identify enrollment in full Medicaid.
▪ Enrollment in Medicare Savings Programs (MSPs)	▪ Enrollment in the Qualified Medicare Beneficiary, Specified Low-Income Medicare Beneficiary (SLMB), and Qualifying Individual (QI) programs (collectively, these comprise the MSPs) for ≥1 month of the year, based on monthly dual eligibility enrollment codes in the MBSF. We used the presence of dual eligibility codes '01', '03' and '06' to identify enrollment in an MSP.
▪ Education	▪ Respondent-reported education in the CAHPS survey
▪ Current smoker	▪ Respondent-reported smoking status in the CAHPS survey

© 2021 Roberts ET et al. *JAMA Health Forum.*

▪ Used helper to complete survey	▪ Captured in the CAHPS survey; proxies for functional impairment
▪ Functional limitations	▪ Respondent-reported difficulty with 1 or more activities of daily living: bathing, dressing, eating, using chairs, walking, and using the toilet.
▪ Self-reported general health and mental health scores	▪ Respondent-rated health on a scale of 1-5, where 1 indicates poor self-rated health or mental health and 5 indicates excellent self-rated general or mental health.

Enrollment in Medicaid or one of the Medicare Savings Programs serve as proxies for socioeconomic status.[24] Baseline means of these covariates for large practices (111-150 clinicians) and small practices (50-89 clinicians) are shown in Table 1 of the main manuscript.

Other covariates

In addition to respondent-level characteristics, we adjusted for annual county-level Medicare Advantage (MA) penetration rates and Hospital Referral Region (HRR) fixed effects. We measured MA penetration using the Area Health Resources File.[25] Adjustment for the MA penetration rate accounts for possible spillovers of the Medicare Advantage program onto care patterns in the fee-for-service Medicare population[26] (the focus of our analyses). Adjustment for HRR fixed effects controls for time-invariant market-level characteristics.

Difference-in-differences model

For each composite and domain-specific patient experience score, we estimated a linear difference-in-differences model of the form:

$$E(Score_{i,t,k,c,h}) = \beta_0 + \beta_1 LargePractice_k + \beta_2(2015 \text{ or } 2016)_t + \beta_3 LargePractice_k \times (2015 \text{ or } 2016)_t + \beta_4 X_{i,t} + \beta_5 MA_{c,t} + year_t + HRR_h \quad (3)$$

where $Score_{i,t,k,c,h}$ is a score for respondent *i* in year *t* who was attributed to practice *k* and lived in county *c* and Hospital Referral Region *h;* $LargePractice_k$ indicates that practice *k* had 111-150 clinicians; $(2015 \text{ or } 2016)_t$ denotes the post-intervention period (2-3 years after the

© 2021 Roberts ET et al. *JAMA Health Forum.*

introduction of the reporting mandate); and $E(.)$ denotes expectation. The model included year fixed effects ($year_t$); respondent characteristics (X_{it}); annual county-level MA penetration rates (MA_{ct}); and HRR fixed effects (HRR_h). Thus, our estimate of β_3 represents the adjusted within-HRR differential change in patient experiences associated with mandatory public reporting for large practices (pooled across HRRs), through 2-3 years after the mandate's introduction. We adjusted all models for survey weights and clustered standard errors at the practice taxpayer identification number (TIN) level.

To facilitate interpretation, we scaled difference-in-differences estimates ($\hat{\beta}_3$) by the practice-level standard deviation of the corresponding patient experience score in the pre-intervention period. We estimated the practice-level standard deviation of each score ($\hat{\sigma}$) by fitting a multilevel linear regression model with practice (TIN) random effects to pre-intervention period survey data and adjusting for the respondent characteristics described above. The resulting scaled estimates and 95% confidence intervals (CIs) are given by:

Scaled estimate = $\hat{\beta}_3/\hat{\sigma}$; 95% CI: $\frac{\hat{\beta}_3}{\hat{\sigma}} \pm 1.96\frac{se(\hat{\beta}_3)}{\hat{\sigma}}$

These scaled estimates can be interpreted as effect sizes relative to the distribution of practice scores in the baseline period. For example, an effect size of -0.16 SDs is equivalent to the difference between the median practice (50th percentile) and a practice at the 44th percentile of performance on a patient experience measure, assuming practice-level scores are normally distributed. Results are in Table 2 of the main manuscript.

Policy context and interpretation of difference-in-differences estimates

The interpretation of our difference-in-differences estimates reflects the structure and phase-in of the PQRS and VM. As described in **eTable 1,** VM payment incentives were fully phased in for both large practices (111-150 clinicians) and smaller practices (50-89 clinicians) around the time large practices were required to begin reporting CAHPS measures for the PQRS. However, the VM was not fully implemented for either large or smaller practices prior to

© 2021 Roberts ET et al. *JAMA Health Forum.*

the phase-in of the reporting mandate in 2014. (While large practices had the option to receive VM payment adjustments based on 2013 performance, very few exercised this option.[27])

Because of this program structure, our difference-in-differences estimates capture the association between mandatory public reporting and patient experiences with care *in the context of pay-for-performance incentives*. These estimates may capture *both* responses to public reporting incentives (independent of pay-for-performance) *and* potential interactions between public reporting and pay-for-performance incentives (e.g., if incentives attributable to public reporting are amplified when practices are also exposed to pay-for-performance incentives).

To formalize this idea, suppose the causal model describing the relationship between these programs and patient care is a function of practice exposure to public reporting requirements, pay-for-performance incentives, and an interaction between these programs. This model can be written as:

$$E(Score_{ikt}|Report, P4P) = \gamma_0 + \gamma_1 Report_{kt} + \gamma_2 P4P_{kt} + \gamma_3(Report_{kt} \times P4P_{kt}) \quad (4)$$

where $Score_{ikt}$ is a score for patient *i* of practice *k* in year *t; $Report_{kt}$* denotes practice *k's* exposure to the reporting mandate in year *t*; and $P4P_{kt}$ denotes practice exposure to pay-for-performance incentives. Patient- and market-level covariates are held constant.

Our empirical difference-in-differences model (model 3) estimates the change in patient experiences from the pre-intervention to the post-intervention periods in large practices that became subject to the reporting mandate (first difference) to contemporaneous changes among smaller unaffected practices (second difference). When there is an interaction between public reporting and pay-for-performance incentives, as written in model (4), our empirical difference-in-differences model gives the following estimates:

<u>Change among large practices that became subject to the reporting mandate</u>:

Post-intervention period: $E(Score_{ikt}|Report = 1, P4P = 1) = \gamma_0 + \gamma_1 + \gamma_2 + \gamma_3$

© 2021 Roberts ET et al. *JAMA Health Forum.*

Pre-intervention period: $E(Score_{ikt}|Report = 0, P4P = 0) = \gamma_0$

<u>First difference</u>: $\gamma_1 + \gamma_2 + \gamma_3$

<u>Change among smaller practices that were unaffected by the reporting mandate</u>:

Post-intervention period: $E(Score_{ikt}|Report = 0, P4P = 1) = \gamma_0 + \gamma_2$

Pre-intervention period: $E(Score_{ikt}|Report = 0, P4P = 0) = \gamma_0$

<u>Second difference</u>: γ_2

<u>Empirical difference-in-differences estimate</u>: $\gamma_1 + \gamma_3$ (5)

Thus, only pay-for-performance incentives that were identical for large and smaller practices in the post-intervention period (γ_2 in model (4)) are held constant in the empirical model. When there is an interaction between program exposures (meaning that the association between public reporting and patient experiences *differs* in the context of pay-for-performance), our empirical estimates capture both γ_1 and γ_3. Here, $\gamma_1 + \gamma_3$ captures changes in patient care associated with the introduction of mandatory public reporting *in the context of context of pay-for-performance incentives.* Given the salience of the pay-for-performance context, this is the interpretation we assign our difference-in-differences estimates. These estimates are relevant to policy because public reporting and pay-for-performance programs are often closely linked, as was the case with the PQRS and VM and continues in the MIPS.

Practice compliance with the CAHPS reporting mandate

We examined practice compliance with the CAHPS reporting mandate by comparing reporting rates among large practices (affected by the mandate) and smaller unaffected practices in 2015-2016 vs. 2014. We found that a higher proportion of large practices reported CAHPS measures in 2015-2016 than did small practices, and that compliance with the mandate increased rapidly among large practices after 2014 **(eFigure 4)**.

To formally compare changes in reporting between large and smaller practices by 2015-2016, we estimated the practice-level linear regression model:

© 2021 Roberts ET et al. *JAMA Health Forum.*

$$E(Report_{kt}) = \delta_0 + \delta_1 LargePractice_{kt} + \delta_2(2015 \text{ or } 2016)_t + \delta_3 LargePractice_{kt} \times (2015 \text{ or } 2016)_t + year_t \quad (6)$$

Above, δ_3 represents the differential increase in public reporting of CAHPS measures among large vs. smaller practices from 2014 to 2015-2016. We estimated a differential increase of 32.3 percentage points (P<0.001), demonstrating that the mandate became an increasingly salient determinant of practice reporting over time. Our difference-in-differences design is predicated on this "first-stage" practice response to the mandate.

Tests of difference-in-differences assumptions

The difference-in-differences design isolates changes in patient experiences with care associated with public reporting, in the context of a pay-for-performance program, under the assumption that differences in patient experiences between large and smaller practices would have remained constant had the reporting mandate among large practices not been implemented. We tested this assumption in two ways.

First, we examined whether estimates could have been biased by differential changes in the composition of patients in large vs. smaller practices over our study period. Such changes could limit our ability to isolate changes in patient experiences with care associated with the reporting mandate from patient-level confounders. To assess this source of this bias, we compared changes in patient characteristics from Table 1 of the main manuscript, among patients of large vs. smaller practices from the pre-intervention to post-intervention periods. Specifically, we estimated a patient-level linear difference-in-differences model for each characteristic, which was analogous to equation (2) but did not adjust for other patient-level covariates. The coefficient on the interaction between the large practice and post-period terms gives the differential change in that characteristic between patients of large vs. smaller practices from the pre- to post-intervention periods. Estimates are in Table 1 of the main manuscript.

© 2021 Roberts ET et al. *JAMA Health Forum.*

Below, **eFigure 3** plots means of selected patient characteristics (age, sex, full Medicaid enrollment, disability status, the CMS-HCC risk score, presence of functional limitations, and indices for self-rated physical and mental health) as a time series for large and smaller practices to identify any systematic compositional changes. As the table and figure show, we found no meaningful changes in the characteristics of patients in large and smaller practices over time, suggesting that bias from compositional changes among patients is unlikely.

As a related check, we examined whether the characteristics of large vs. smaller practices changed differentially from the pre- to post-intervention periods of our analysis. We examined changes in: (1) the number of clinicians billing under a practice's taxpayer identification number (TIN); (2) the proportion of clinicians billing under the practice TIN who were primary care clinicians; (3) the proportion of clinician billing under the practice TIN who were vertically integrated with a hospital (based on billing patterns in Medicare claims).[28] These variables capture important practice attributes related to the mix of clinicians and organizational structure that could have affected performance on CAHPS measures. We did not find statistically significant differential changes between large vs. smaller practices across the pre- and post-intervention study periods (**eTable 8**). These findings support the assumption that the difference-in-differences design isolates changes in patient experiences associated with mandatory public reporting.

Second, we tested the difference-in-differences assumption that differences in patient experience scores of large vs. smaller practices would have remained constant in the absence of the public reporting mandate for large practices. We evaluated the plausibility of this assumption by comparing pre-intervention trends in patient experience scores among large vs. smaller practices from 2011-2013. Finding "parallel" pre-intervention trends between large and smaller groups of practices (equivalent to constant pre-intervention differences) suggests that between-group differences likely would have remained constant in the absence of the reporting

© 2021 Roberts ET et al. *JAMA Health Forum.*

mandate.[29] To compare pre-intervention trends between large and smaller practices, we estimated patient-level linear event-study models for the form:

$$E(Score_{i,t,k,c,h}) = \alpha + \varphi LargePractice_k + \sum_{t\neq 2013}\theta_t\,(year = t) + \sum_{t\neq 2013}\delta_t\,(year = t) \times LargePractice_k + \beta_4 X_{i,t} + \beta_5 MA_{c,t} + year_t + HRR_h \quad (7)$$

We estimated separate models for each composite and domain-level patient experience score. In each model, we omitted patient experiences in 2013 as the reference period (outcomes in the 2014 transition year are excluded). We plotted $\hat{\delta}_t$, which are estimates of annual differential changes between large vs. smaller practices (relative to differences between large vs. smaller practices in 2013), and associated 95% confidence intervals, in **eFigure 5.** As shown in the plots, estimates of $\hat{\delta}_t$ are close to and statistically indistinguishable from 0 for *t*=2011 and *t*=2012, implying constant pre-intervention differences (parallel pre-intervention trends).

eFigure 5 also traces out time-varying differential changes past 2013 (captured by $\hat{\delta}_{2016}$ and $\hat{\delta}_{2015}$), which we used to examine whether changes in care associated with mandatory public reporting for large practices emerged over time. We found no evidence of differential improvements in patient experiences emerging by 2016.

Sensitivity analyses

We conducted two sensitivity analyses. First, we analyzed changes in patient experiences with care from 2011-2013 to 2015-2016 using data from concurrent Fee-for-service Medicare CAHPS surveys (i.e., surveys administered from 2011-2013 and 2015-2016). We attributed patients to practices based on concurrent-year primary care claims to be consistent with the period in which we measured patient experiences. Our results, shown in **eTable 9**, were not substantively different from our main analyses in Table 2 of the main manuscript.

Second, we re-ran our difference-in-differences models on a broader sample of 26,380 Fee-for-service Medicare CAHPS respondents whom we attributed to practices based on

© 2021 Roberts ET et al. *JAMA Health Forum.*

outpatient visits with primary care clinicians or specialists. (Our main analyses only included respondents attributed to practices based on visits with primary care clinicians.) This broader sample was approximately 20% larger than our main sample, though it was generally comparable on measured health risks: for example, the mean Medicare HCC risk score among patients in the larger sample was 1.25, versus 1.26 in our main sample. Furthermore, only slightly higher proportions of respondents in the broader sample had full or partial Medicaid (6.9% with full Medicaid and 4.0% with partial Medicaid) compared with our main sample (6.3% with full Medicaid and 3.6% with partial Medicaid). Results of difference-in-differences models run on this broader sample, reported in **eTable 10,** closely resembled our main findings.

© 2021 Roberts ET et al. *JAMA Health Forum.*

eFigure 3: Means or proportions of patient characteristics in large vs. smaller practices from 2011-2013 and 2015-2016

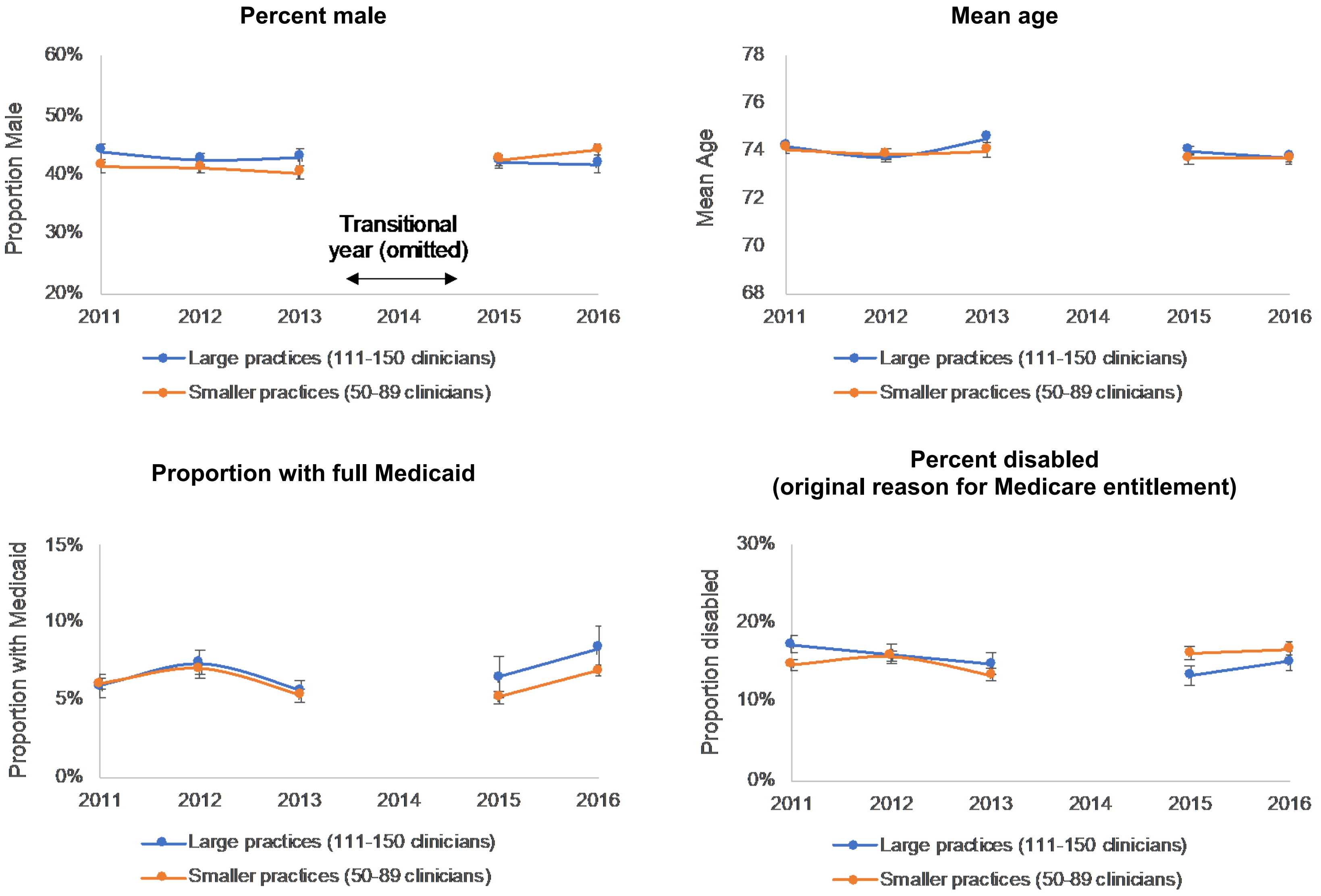

© 2021 Roberts ET et al. *JAMA Health Forum.*

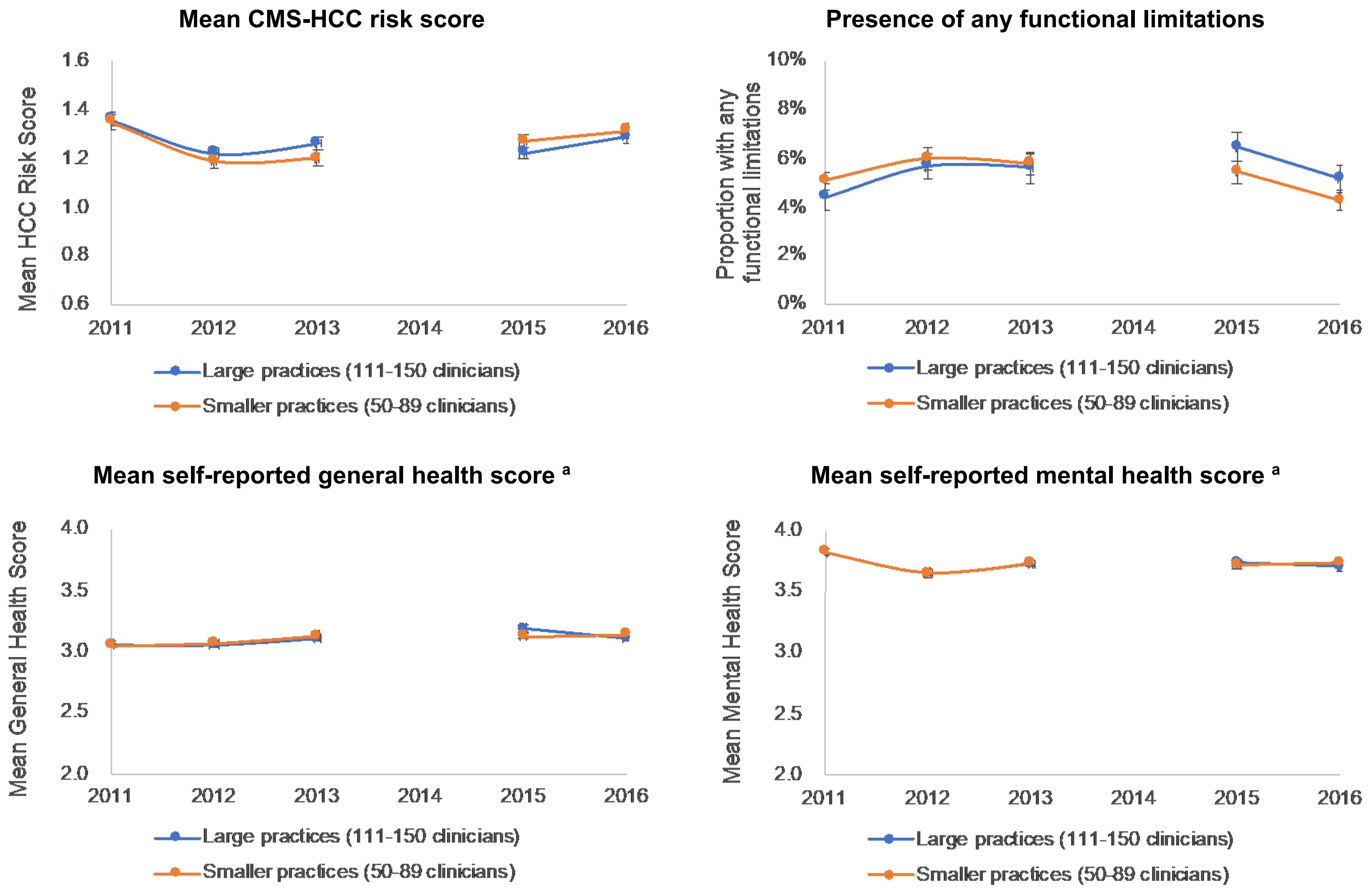

Plotted are annual means or proportions of patient characteristics among patients attributed to large practices (111-150 clinicians) vs. smaller practices (50-89 clinicians). Patient characteristics are measured in the year in which we attributed patients to practices. Error bars around the point estimates denote 95% confidence intervals. See the notes to Table 1 of the main manuscript for variable definitions.
[a] Scale of 1 to 5, where 1 indicates poor self-rated health or mental health and 5 indicates excellent self-rated general or mental health.

© 2021 Roberts ET et al. *JAMA Health Forum.*

eFigure 4: Proportions of large and smaller practices publicly reporting CAHPS measures in 2014 vs. 2015-2016

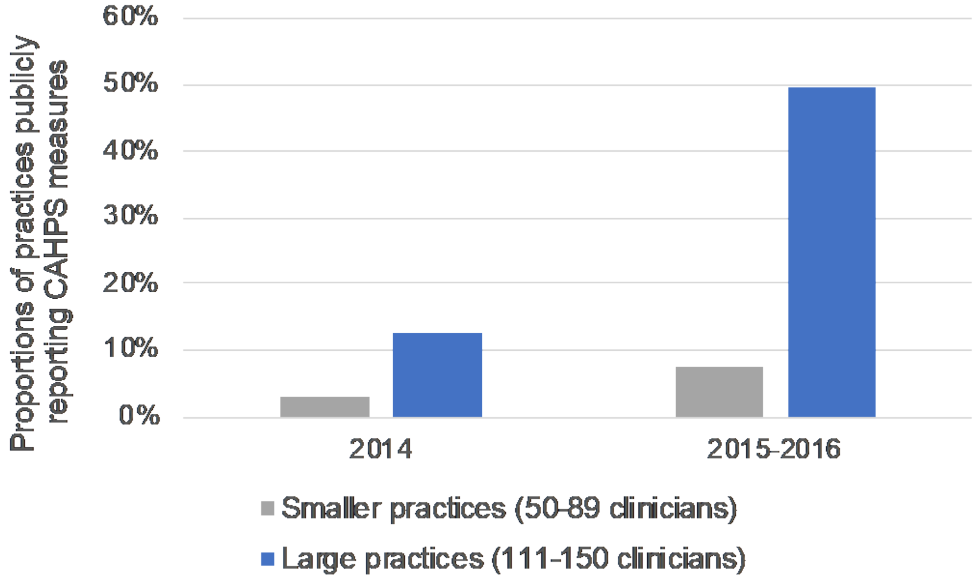

This graph shows proportions of practices publicly reporting CAHPS measures, based on practice size and reporting data in VM Practice Files from 2014-2016. Large practices with ≥100 clinicians were required to publicly report CAHPS measures starting in 2014.

© 2021 Roberts ET et al. *JAMA Health Forum.*

eFigure 5: Event-study plots of annual differential changes in composite and domain-level patient experience scores between large and smaller practices (relative to 2013)

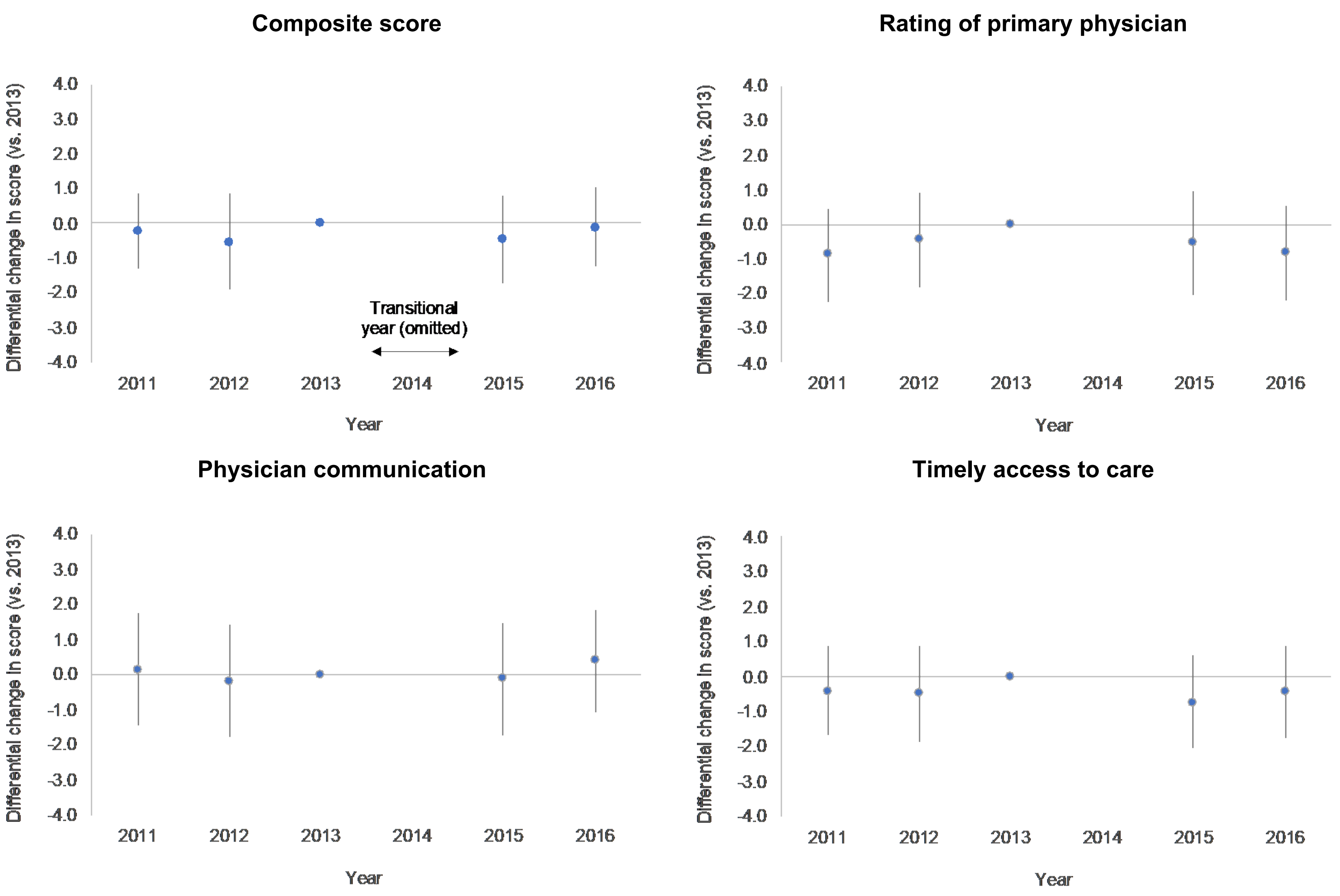

© 2021 Roberts ET et al. *JAMA Health Forum.*

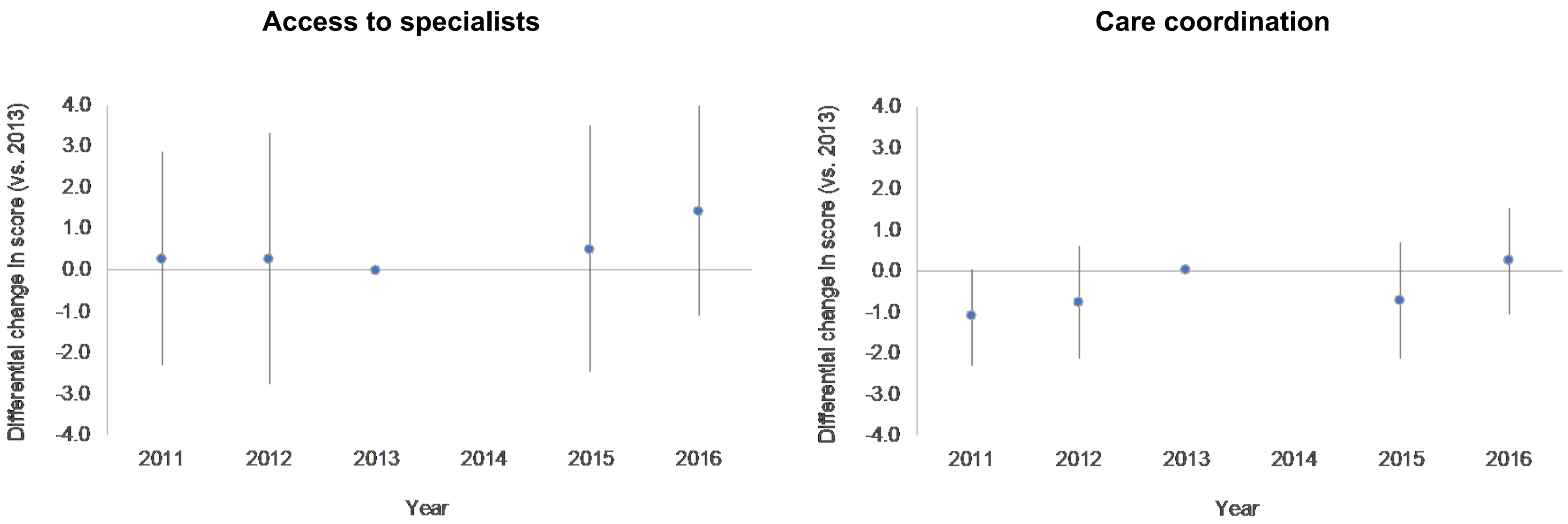

Event study plots show the differential change in composite or domain-specific patient experience scores between large practices (111-150 clinicians) and smaller practices (50-89 clinicians) by year relative to 2013. We omitted 2014 as a transitional year. Patient experience scores reported on a 0-100 scale (to which we standardized responses from all survey items). The shaded blue circles represent the estimates $\hat{\delta}_t$ from Equation 6 and the error bars represent 95% confidence intervals for these estimates. All estimates adjusted for respondent characteristics from Table 1 of the main manuscript, annual county-level MA penetration rates, HRR fixed effects, year fixed effects, and for survey weights. The 95% confidence intervals were calculated using robust standard errors clustered at the practice (taxpayer identification number) level.

© 2021 Roberts ET et al. *JAMA Health Forum.*

eTable 8: Pre-intervention practice characteristics and changes in the characteristics of large vs. smaller practices in difference-in-differences analysis

	Pre-intervention period (2011-2013)		Change from pre-intervention to post-intervention period (2011-2013 to 2015-2016)				
					Differential change [c]		
**Practice-level characteristics:** [a]	Large practices [b] (111-150 clinicians)	Smaller practices [b] (50-89 clinicians)	Large practices (111-150 clinicians)	Smaller practices (50-89 clinicians)	Estimate	95% CI	P-value
Number of clinicians billing under practice TIN	130.3	66.4	0.2	0.5	-0.3	(-2.7, 2.0)	0.79
Proportion of clinicians in practice TIN who are primary care clinicians, % [d]	51.6	53.9	-0.1	0.2	-0.3	(-0.7, 0.1)	0.13
Proportion of clinicians in practice TIN vertically integrated with a hospital, % [e]	21.6	12.2	-5.2	-1.2	-4.0	(-8.1, 0.1)	0.06

[a] Estimates based on 2,186 practice-years for large practices (111-150 clinicians) and smaller practices (50-89 clinicians) in period 2011-2013 and 2015-2016.
[b] Practice size calculated as the number of unique clinicians that billed under a practice's taxpayer identification number in the year prior to the survey.
[c] That is, changes in the characteristic shown in the table row between large and smaller practices from the pre-intervention to the post-intervention periods. We estimated these differential changes by fitting a practice-level linear difference-in-differences model for each characteristic as a function of a post-intervention period indicator, an indicator that a patient's practice had 111-150 clinicians, and an interaction between these indicators. Differential changes are given by the regression coefficient on the interaction term.
[d] Primary care clinicians defined as clinicians with Medicare specialty codes for general practice, family practice, internal medicine, geriatric medicine, nurse practitioner, or physician assistant (specialty codes: 01, 08, 11, 38, 50, 97).
[e] Proportion of clinicians billing >75% of outpatient claims in a hospital outpatient department (place of service code 22) vs. a physician office. We measured the proportion of clinicians whose billing in a hospital outpatient department exceeded this 75% threshold, aggregated to the level of the practice taxpayer identification number and year.

© 2021 Roberts ET et al. *JAMA Health Forum.*

eTable 9: Difference-in-differences estimates based on responses to concurrent Fee-for-service Medicare CAHPS surveys

		Pre-intervention period (2011-2013)			Difference-in-differences estimates [d]			
**Patient experience scores [a]**		Mean scores among large practices (111-150 clinicians) [b]	Standard deviation (SD) of practice scores [c]		Regression Estimate	Effect size (SDs) [e]	95% CI (SDs) [e,f]	P-value [f]
Composite score		80.1	1.6		0.30	0.19	(-0.35, 0.72)	0.50
Domain-specific scores:								
Rating of primary physician		90.6	1.6		0.21	0.13	(-0.48, 0.74)	0.68
Physician communication		88.4	1.6		0.90	0.58	(-0.10, 1.25)	0.09
Timely access to care		66.0	2.4		0.55	0.23	(-0.18, 0.64)	0.27
Access to specialists		84.9	2.7		-0.08	-0.03	(-0.75, 0.69)	0.93
Care coordination		81.5	1.4		0.78	0.55	(-0.12, 1.21)	0.11

[a] Patient experiences with care assessed from the Fee-for-service Medicare CAHPS survey. In this sensitivity analysis, we used responses to surveys administered from 2011-2013 and 2015-2016 to assess patient experiences with care in the same years. We omitted 2014 as a transitional year.
[b] Mean scores among practices with 111-150 clinicians in the pre-intervention period, adjusted for respondent characteristics in Table 1 of the main manuscript, annual county-level MA penetration rates, HRR fixed effects, year fixed effects, and survey weights. Scores are standardized to a 0-100 scale, with higher scores representing better patient experiences with care.
[c] Standard deviation (SD) of the practice-level distribution of patient experience scores, estimated among all practices in the pre-intervention period. For this analysis, we estimated the practice-level standard deviation of scores from CAHPS surveys administered from 2011-2013.
[d] Difference-in-differences estimates represent the differential change in composite or domain-specific patient experience scores between large practices (111-150 clinicians) and smaller practices (50-89 clinicians) from the pre-intervention period (2011-2013) to the post-intervention period (2015-2016), adjusted for respondent characteristics in Table 1 of the main manuscript and survey weights.
[e] Effect sizes are difference-in-differences estimates scaled by the practice-level standard deviation (SD) of each score. The corresponding 95% confidence intervals are also scaled by the practice-level SD in each score.
[f] 95% confidence intervals and P-values were calculated using robust standard errors clustered at the practice (taxpayer identification number) level.

© 2021 Roberts ET et al. *JAMA Health Forum.*

eTable 10: Difference-in-differences estimates among patients attributed to practices based on outpatient claims with primary care clinicians or specialists

		Pre-intervention period (2011-2013)			Difference-in-differences estimates [d]			
**Patient experience scores [a]**		Mean scores among large practices (111-150 clinicians) [b]	Standard deviation (SD) of practice scores [c]		Regression Estimate	Effect size (SDs) [e]	95% CI (SDs) [e,f]	P-value [f]
Composite score		80.2	1.5		-0.01	-0.01	(-0.58, 0.57)	0.98
Domain-specific scores:								
Rating of primary physician		89.7	0.8		-0.03	-0.04	(-1.28, 1.20)	0.95
Physician communication		88.4	1.4		0.19	0.14	(-0.64, 0.93)	0.72
Timely access to care		66.5	2.4		0.01	0.00	(-0.39, 0.39)	0.99
Access to specialists		84.0	2.5		0.58	0.24	(-0.53, 1.01)	0.55
Care coordination		79.8	1.3		0.89	0.68	(-0.06, 1.41)	0.07

[a] Patient experiences with care assessed from the Fee-for-service Medicare CAHPS survey. We used responses to surveys administered from 2012-2014 and 2016-2017 to assess patient experiences with care from 2011-2013 and 2015-2016, respectively. We omitted the 2015 survey, pertaining to patient experiences in 2014, as a transitional year. This sensitivity analysis was based on 26,380 respondents attributed to practices based on outpatient visits with primary care clinicians or specialists.
[b] Mean scores among practices with 111-150 clinicians in the pre-intervention period, adjusted for respondent characteristics in Table 1 of the main manuscript, annual county-level MA penetration rates, HRR fixed effects, year fixed effects, and survey weights. Scores are standardized to a 0-100 scale, with higher scores representing better patient experiences with care.
[c] Standard deviation (SD) of the practice-level distribution of patient experience scores, estimated among all practices in the pre-intervention period. For this analysis, we estimated the practice-level standard deviation of scores from CAHPS surveys administered from 2011-2013.
[d] Difference-in-differences estimates represent the differential change in composite or domain-specific patient experience scores between large practices (111-150 clinicians) and smaller practices (50-89 clinicians) from the pre-intervention period (2011-2013) to the post-intervention period (2015-2016), adjusted for respondent characteristics in Table 1 of the main manuscript and survey weights.
[e] Effect sizes are difference-in-differences estimates scaled by the practice-level standard deviation (SD) of each score. The corresponding 95% confidence intervals are also scaled by the practice-level SD in each score.
[f] 95% confidence intervals and P-values were calculated using robust standard errors clustered at the practice (taxpayer identification number) level.

© 2021 Roberts ET et al. *JAMA Health Forum.*

REFERENCES

1. *2015 Value-Based Payment Modifier Program Experience Report.* Baltimore, MD: Centers for Medicare and Medicaid Services; June 15, 2015 2015.

2. *2016 Value-Based Payment Modifier Program Experience Report.* Baltimore, MD: Centers for Medicare and Medicaid Services; April 2017 2017.

3. *2017 Value-Based Payment Modifier Program Experience Report.* Baltimore, MD: Centers for Medicare and Medicaid Services; September 2017 2017.

4. *2018 Value-Based Payment Modifier Program Experience Report.* Baltimore, MD: Centers for Medicare and Medicaid Services; July 2018 2018.

5. *2013 Physician Quality Reporting System (PQRS): 2015 PQRS Payment Adjustment.* Baltimore, MD: Centers for Medicare and Medicaid Services; August 2013 2013.

6. Dowd BE, Swenson T, Parashuram S, Coulam R, Kane R. PQRS Participation, Inappropriate Utilization of Health Care Services, and Medicare Expenditures. *Med Care Res Rev.* 2016;73(1):106-123.

7. *2015 Reporting Experience Including Trends (2007-2015): Physician Quality Reporting System.* Baltimore, MD: Centers for Medicare and Medicaid Services;2017.

8. Chapter 2: Medicare’s new framework for paying clinicians. In: *Report to the Congress: Medicare and the Health Care Delivery System.* Washington DC: Medicare Payment Advisory Commission; 2016:29-54.

© 2021 Roberts ET et al. *JAMA Health Forum.*

9. Koltov MK, Damle NS. Health policy basics: physician quality reporting system. *Ann Intern Med.* 2014;161(5):365-367.

10. PQRS CAHPS Frequently Asked Questions. Press Ganey Associates. https://helpandtraining.pressganey.com/lib-docs/default-source/Government-Initiatives/pqrs-cahps-faq.pdf?sfvrsn=4. Published 2015. Accessed March 31, 2020.

11. Chen LM, Epstein AM, Orav E, Filice CE, Samson L, Joynt Maddox KE. Association of practice-level social and medical risk with performance in the medicare physician value-based payment modifier program. *JAMA.* 2017;318(5):453-461.

12. Medicare program; revisions to payment policies under the physician fee schedule, clinical laboratory fee schedule & other revisions to Part B for CY 2014. Final rule with comment period. *Fed Regist.* 2013;78(237):74449-74452.

13. *Detailed Methodology for the 2018 Value Modifier and the 2016 Quality and Resource Use Report.* Baltimore, MD: Centers for Medicare and Medicaid Services; September 2017 2017.

14. Mugge A. Physician Quality Reporting System (PQRS). Medicare Learning Network Connects. Published Undated. Accessed March 31, 2020.

15. *Medicare Advantage, Medicare Part D, and Medicare Fee-For-Service Consumer Assessment of Healthcare Providers and Systems (CAHPS) Survey: Supporting Statement Part B.* Baltimore, MD: Centers for Medicare and Medicaid Services; February 2, 2021 2021.

© 2021 Roberts ET et al. *JAMA Health Forum.*

16. Moen EL, Bynum JPW. Evaluation of Physician Network-Based Measures of Care Coordination Using Medicare Patient-Reported Experience Measures. *J Gen Intern Med.* 2019;34(11):2482-2489.

17. McWilliams JM, Landon BE, Chernew ME, Zaslavsky AM. Changes in Patients' Experiences in Medicare Accountable Care Organizations. *New England Journal of Medicine.* 2014;371(18):1715-1724.

18. Roberts ET, Mehrotra A, McWilliams JM. High-Price And Low-Price Physician Practices Do Not Differ Significantly On Care Quality Or Efficiency. *Health Affairs.* 2017;36(5):855-864.

19. O'Malley AJ. Weak correlations in health services research: Weak relationships or common error? *Under review.* 2020.

20. *Medicare Shared Savings Program Interaction with the 2017 Value Modifier: Frequently Asked Questions.* Baltimore, MD: Centers for Medicare and Medicaid Services; September 2016 2016.

21. Medicare Program; Revisions to Payment Policies Under the Physician Fee Schedule, Clinical Laboratory Fee Schedule, Access to Identifiable Data for the Center for Medicare and Medicaid Innovation Models & Other Revisions to Part B for CY 2015. 2014;79(219):67931-67941.

22. Research Triangle Institute (RTI) Race Code. Research Data Assistance Center. https://resdac.org/cms-data/variables/research-triangle-institute-rti-race-code. Accessed July 30, 2021.

© 2021 Roberts ET et al. *JAMA Health Forum.*

23. Evans MA, Pope GC, Kautter J, et al. Evaluation of the CMS-HCC Risk Adjustment Model. *CfMM Services, Editor.* 2011.

24. Samson LW, Finegold K, Ahmed A, Jensen M, Filice CE, Joynt KE. Examining measures of income and poverty in medicare administrative data. *Medical care.* 2017;55(12):e158-e163.

25. Area Health Resources File (AHRF). In: Administration HRaS, ed. 2019-2020 Release ed. Rockville, MD.

26. Glied S, Zivin JG. How do doctors behave when some (but not all) of their patients are in managed care? *J Health Econ.* 2002;21(2):337-353.

27. Performance And Participation Of Physicians In Year One Of Medicare's Value-Based Payment Modifier Program. *Health Affairs.* 2017;36(12):2175-2184.

28. Neprash HT, Chernew ME, Hicks AL, Gibson T, McWilliams JM. Association of Financial Integration Between Physicians and Hospitals With Commercial Health Care Prices. *Jama Internal Medicine.* 2015;175(12):1932-1939.

29. Daw JR, Hatfield LA. Matching and regression to the mean in difference-in-differences analysis. *Health services research.* 2018;53(6):4138-4156.

© 2021 Roberts ET et al. *JAMA Health Forum.*
